# Age‐Related Dynamics and Spectral Characteristics of the TCRβ Repertoire in Healthy Children: Implications for Immune Aging

**DOI:** 10.1111/acel.14460

**Published:** 2025-01-02

**Authors:** Mingyan Fang, Yu Miao, Lin Zhu, Yunpeng Mei, Hui Zeng, Lihua Luo, Yuan Ding, Lina Zhou, Xueping Quan, Qin Zhao, Xiaodong Zhao, Yunfei An

**Affiliations:** ^1^ BGI Research Shenzhen China; ^2^ School of Life Sciences Lanzhou University Lanzhou Gansu Province China; ^3^ Henan Academy of Sciences Zhengzhou China; ^4^ College of Life Sciences University of Chinese Academy of Sciences Beijing China; ^5^ Department of Child Health Care Children's Hospital of Chongqing Medical University Chongqing China; ^6^ National Clinical Research Center for Child Health and Disorders, Ministry of Education Key Laboratory of Child Development and Disorders Chongqing Key Laboratory of Child Infection and Immunity Chongqing China; ^7^ Department of Rheumatology and Immunology Children's Hospital of Chongqing Medical University Chongqing China; ^8^ Department of Endocrinology Children's Hospital of Chongqing Medical University Chongqing China

**Keywords:** aging, T‐cell receptor, TCRβ repertoire

## Abstract

T‐cell receptor (TCR) diversity is crucial for adaptive immunity, yet baseline characterizations in pediatric populations remain sparse. We sequenced the TCRβ chain of 325 healthy Chinese children aged 0–18, categorized into six age groups. We also analyzed cellular composition and TCRβ associations using flow cytometry in 81 of these samples. Our results indicate a decrease in TCRβ diversity with age, characterized by an increase in high‐frequency clonotypes and notable changes in CDR3 length and V(D)J gene usage. These changes are influenced by early life vaccinations and antigen exposures. Additionally, we found a significant association between reduced TCRβ diversity and a decrease in CD4^+^ T naïve cells. We also developed a predictive model that identifies specific TCRβ features as potential biomarkers for biological age, validated by their significant correlation with changes in the immune repertoire. These findings enhance our understanding of age‐related variations in the TCRβ repertoire among children, providing resourceful information for research on pediatric TCR in health and disease.

## Graphical abstract

Developmental dynamics of TCRβ diversity in Chinese children. We analyzed TCRβ repertoire diversity in 325 healthy Chinese children (0–18 years) through TCRβ sequencing and flow cytometry analysis (*n* = 81). Our analysis revealed the following: Age‐dependent decline in TCRβ diversity characterized by increased high‐frequency clonotypes, altered CDR3 length and V(D)J usage patterns. Correlation between TCRβ diversity reduction and CD4^+^ naive T‐cell depletion. TCRβ features as potential immune age biomarkers. This study establishes baseline characteristics of pediatric TCRβ repertoire development.

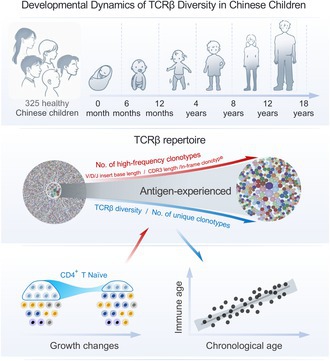

## 1 Introduction

The immune repertoire (IR), comprising the diverse T‐cell receptor (TCR) and B‐cell receptor (BCR), is crucial for adaptive immunity. Its functionality, shaped by TCR and BCR variability, is pivotal in responding to pathogens and immune surveillance against malignancies. This diversity provides valuable insights into individual adaptive immune responses (Liu and Wu 2018).

Antigen receptor diversity stems from the recombination of variable (V), diversity (D), and joining (J) gene segments (Liu and Wu 2018; Zhang et al. 2015), further amplified by junctional diversity. This includes non‐templated nucleotide additions by terminal deoxynucleotidyl transferase (TdT) in V–D and D–J junctions, deletions facilitated by ARTEMIS (IJspeert et al. 2016; Wong et al. 2017), and the involvement of DNA‐dependent protein kinase (DNA‐PK) in the repair processes. These recombination processes, characterized by variable insertions and deletions at V, D, and J junctions, contribute to significant sequence diversity.

By using multiplex PCR or 5′RACE to specifically target the complementary determining region 3 (CDR3) and combining it with high‐throughput sequencing (Wu et al. 2020), researchers can analyze TCR diversity and compare it between diseased and healthy groups. The methodology has been applied in research on various disorders, including allergies (Cao et al. 2020), immunodeficiencies (Fang et al. 2022; Lee et al. 2016), autoimmune diseases (Gomez‐Tourino et al. 2017; Liu et al. 2019), and malignancies (Zhang et al. 2017), providing a comprehensive assessment of the immune system's diversity. This approach uncovers the intricate relationship between the IR and specific diseases, highlighting the emergence of disease‐specific TCRs or BCRs as potential biomarkers (Fang et al. 2022; Liu et al. 2019; Miao et al. 2023). These insights offer new possibilities in the realms of disease prediction, early diagnosis, and the development of innovative therapeutic strategies.

Understanding the characteristics of the IR in normal individuals is crucial for establishing a baseline to identify IR abnormalities, which is instrumental for disease diagnosis and treatment assessment. A recent study employing single‐cell TCR/BCR‐seq to analyze subpopulations of immune cells in the peripheral blood of 166 healthy individuals aged 25–85 underscored an age‐associated functional shift in immune homeostasis, revealing significant changes in 12 immune cell subtypes (Terekhova et al. 2023). This discovery aligns with existing literature on the evolving nature of TCR repertoires and the age‐related decline in diversity (Britanova et al. 2016, 2014; Yager et al. 2008; Nikolich‐Zugich 2018, 2008). Previous research on 1075 healthy children also highlighted a strong correlation between age and lymphocyte counts, especially in T‐cells (Ding et al. 2018), further emphasizing the importance of understanding the relationship between TCRβ characteristics and age. Such investigation, including the exploration of the dynamics of immune system, immune cell populations, and their counts, is crucial for deepening our understanding of immune responses across different age groups in health and disease contexts.

Although significant research has been conducted on the IR characteristics of adults over 18 (Sun et al. 2022; Lian et al. 2019; Song et al. 2022), there is a noticeable scarcity of studies on healthy children, especially those aged 0–6 (Britanova et al. 2016, 2014; Lian et al. 2019; Emerson et al. 2017; van de Sandt et al. 2023). This disparity highlights a significant knowledge gap in understanding how the IR develops and changes from infancy through adolescence. Consequently, there is a pressing need for more comprehensive examinations of IR profiles spanning the entire age range of 0–18 years, to better understand pediatric immune development and its implications for health and disease.

Given the limited understanding of IR development in children, our study aimed to bridge this gap by focusing on the variability of the TCR repertoire in relation to age and gender. We examined a cohort of 325 healthy Chinese children aged 0–18 years, finding a marked decrease in TCRβ diversity with advancing age, showing a significant negative correlation. Additionally, we observed intricate variations in the utilization of VJ genes and in the patterns of insertions and deletions across different age groups. These characteristics could serve as robust predictors of donor age. To our knowledge, this research is the most comprehensive exploration of the TCR repertoire in the 0–18 age demographic, offering foundational insights into how TCR development correlates with age.

## 2 Methods

### 2.1 Sample Collection

In this study, initial sample collection was carried out through a multicenter approach involving major hospitals across China (Chongqing, Beijing, Shanghai, and Shenzhen), which allowed for a diverse and representative sampling across various geographic and demographic segments. A total of 346 samples were systematically randomized from a pool of 1075 healthy children across a balanced distribution of age and gender, ranging from 0 to 18 years, to create a representative dataset of peripheral blood lymphocyte subsets within the Chinese pediatric population (Ding et al. 2018). All participants underwent comprehensive health screenings to confirm their status as “healthy.” Criteria for inclusion encompassed a normal nutritional status, absence of fever, no recent medication use, and no recent contact with infectious diseases. Additionally, eligible children had not received immunizations or blood transfusions within the last 4 weeks and had no history of atopic, infectious, hematological, or immunological disorders. These stringent criteria ensured that only individuals meeting the highest standards of health were included in our study. Flow cytometry data were available for a subset of 81 samples, which had previously been utilized to characterize the peripheral blood lymphocyte profiles in detail. The study received ethical approval from the Institutional Review Boards (IRBs) at the Children's Hospital of Chongqing.

### 2.2 TCR Repertoire Sequencing

The CDR3 sequences of TCRβ genes were amplified using previously described multiplex primers (Wu et al. 2020) and two complete sequencing adapter primers from equal amounts of DNA samples (1.2 μg) across all subjects. The amplicons underwent purification followed by a second round of amplification, consistent with previous studies (Cao et al. 2020; Fang et al. 2022). The resultant libraries were sequenced on MGIseq (MGI, Shenzhen, China) platform with 200 bp single‐end reads.

### 2.3 TCR Sequencing Data Analysis Using IMonitor

In‐depth analysis and mapping of TCR sequencing data were performed using IMonitor software (Zhang et al. 2015). After quality control, V(D)J alignment against the IMGT database (https://www.imgt.org/) was executed using BLAST. To standardize the dataset and mitigate sequencing depth variability, 1 M (1 × 10^6^) clonotypes were randomly selected from the CDR3 clonotypes pool in each sample. Samples with outlier values for “Unique_clonotype” within each age category were excluded to reduce biases from sequencing depth and atypical samples.

Subsequent computations yielded immune indicators for the TCRβ repertoire across all samples. In the final statistical evaluation, conducted with IMonitor (Zhang et al. 2015), some economic indices were introduced to generate metrics to measure repertoire diversity and to evaluate repertoire homogeneity, encompassing clonal abundance, Shannon diversity index for the clonal repertoire, Pielou's evenness index, frequency of the top 100 clonotypes, count of predominant clonotypes, and a composite measure of Shannon diversity and Pielou's evenness for V/J gene usage. The formulas for the metrics that involve calculations are as follows (Zhang et al. 2015; Chiffelle et al. 2020):

$${\text{Shannon}}^{\prime }s\;H=-\sum_{i=1}^{N}p\left(i\right)\log_{2}p\left(i\right)$$

$${\text{Pielou}}^{\prime }s\;\text{evenness}=\frac{\sum_{i=1}^{N}p\left(i\right)\log_{2}p\left(i\right)}{\log_{2}\left(N\right)}$$

$$\text{Gini skewing index}=1-\sum_{i=1}^{N}p{\left(i\right)}^{2}$$

where *p*(*i*) is the frequency of CDR3 sequence *i* in the repertoire and *N* is the total number of unique sequences.

Clonotype CR4: The cumulative frequency of the top 4 clonotypes is simply the sum of the frequencies of the top 4 most abundant clonotypes.

In our analysis, TCR rearrangements were classified into three categories: “in‐frame,” “out‐of‐frame,” and “nonfunction.” The “out‐of‐frame” category encompasses rearrangements that either contain a premature stop codon (“out‐of‐frame [Stop_Codon]”) or whose sequence length is not divisible by three (“out‐of‐frame [CDR3_length]”), both indicative of frameshift mutations. Such alterations typically result in nonfunctional proteins. Additionally, “nonfunction” refers to rearrangements involving V and J genes identified as pseudogenes in the IMGT database, which are generally unable to produce functional proteins due to genetic anomalies. In contrast, “in‐frame” sequences do not exhibit these disruptive features and are thus considered potentially capable of producing functional TCR clonotypes.

### 2.4 Evaluation of TCRβ Repertoire Similarity Between Samples

In our analysis, we employed the Jaccard coefficient to assess the similarity of TCRβ repertoires across samples, taking into account the abundance of each clonotype. In this approach, the Jaccard coefficient is calculated by dividing the sum of abundances for clonotypes present in both samples by the sum of abundances for all clonotypes across the samples. Specifically, if ***I*** represents the set of clonotypes common to both samples and ***U*** represents the set of all clonotypes across the samples, the numerator of the coefficient is the sum of the combined counts ($a_{i}+b_{i}$) for each clonotype i in ***I*** and the denominator is the sum of the combined counts for each clonotype in ***U***. This method provides a more nuanced metric of similarity, reflecting not only the presence of shared clonotypes but also their abundance, thereby offering a comprehensive view of the TCRβ repertoire overlap.

$$J=\frac{\sum_{i\in I}\left(a_{i}+b_{i}\right)}{\sum_{i\in U}\left(a_{i}+b_{i}\right)}$$

where ***I*** represents the set of clonotypes that are present in both samples. ***U*** represents the set of all clonotypes across both samples. $a_{i}$ represents the count of clonotype i in sample A. $b_{i}$ represents the count of clonotype i in sample B.

### 2.5 Annotation of Pathology‐Associated TCR Sequence

To annotate the pathology‐associated TCR sequences, all TCR clonotypes identified within the sample cohort were cross‐referenced against three established public TCR databases. These databases include VDJdb (https://vdjdb.cdr3.net/) (Shugay et al. 2018), Pan immune repertoire database (PIRD, https://db.cngb.org/pird/) (Zhang et al. 2020), and McPAS‐TCR (http://friedmanlab.weizmann.ac.il/McPAS‐TCR/) (Tickotsky et al. 2017), which collectively provide a comprehensive repository of known TCR sequences implicated in various pathological states.

### 2.6 Identifying and Defining Public Clonotypes Through Stratified Random Sampling

The concept of “public clonotypes” was defined based on the cohort with the smallest sample size, specifically the 0–6‐month group with 28 samples. For each age cohort, we conducted stratified random sampling of 28 samples, repeating this procedure 100 times. The mean number of public clonotypes was calculated from these iterations. Public clonotypes were identified based on their prevalence in more than half of these random samples, focusing on clonotypes that were unique to each iteration.

### 2.7 Characterization of Public Clonotypes Across Age Cohorts

We analyzed the sequence similarity of public clonotypes across different age cohorts using the clustringr package (version 1.0) in R. CDR3 sequences with a Levenshtein distance of ≤ 2 were considered highly similar and thus categorized together.

For each age‐specific cohort, a subset equal in number to the public clonotype count was randomly selected. The CDR3 lengths of these clonotypes were then compared with those of the identified public clonotypes.

### 2.8 Validation of TCR Repertoire Characteristics Using Independent Dataset

To validate whether the public clonotypes identified in our cohort are universal across genetically diverse populations, we analyzed publicly available TCRβ repertoire sequencing data from the immuneACCESS database. The validation dataset consisted of 100 blood samples collected at four time points from 25 healthy individuals aged 0–20 years (Mitchell et al. 2022). We categorized these samples according to our study's established age groups based on collection time. To avoid redundancy, we retained only one dataset per individual within each age group. Due to limited sample size in the 12–18 years category, we included two additional samples (ages 19.45 and 19.84 years) in this group, resulting in a total of 74 unique samples.

Given the variation in sequencing depths across samples (average ~150,000 productive clonotypes per sample), we standardized the dataset by randomly subsampling 100,000 clonotypes from each sample. We then recalculated key IR metrics, including the number of unique clonotypes, Shannon diversity index, Gini coefficient, percentage of top 100 clonotypes, and number of high‐frequency clonotypes.

To evaluate whether the public clonotypes identified in our cohort are region specific or more universally distributed across genetically diverse populations, we analyzed the frequency of shared clonotypes in the validation dataset. Consistent with our primary cohort methodology, we defined public clonotypes as those shared by more than 50% of samples within each age group.

### 2.9 Principal Component Regression Analysis

To assess the relationship between TCRβ repertoire features and biological age, we conducted Pearson correlation analyses on various TCRβ characteristics to determine their correlations with age across all samples. The dataset was partitioned into a training set, comprising 70% of the samples, and a testing set, which included the remaining 30%. Principal component regression was then applied to the training set using the identified TCRβ features to construct an age prediction model. The model's predictive accuracy was gauged by correlating the predicted immune age with the actual biological age. The model was further validated by applying it to the testing dataset to predict ages and assess its performance. Additionally, the significance of each variable within the model was analyzed to determine the primary predictors of immune age.

### 2.10 Ridge Regression Analysis

To identify the primary T‐cell subsets affecting TCRβ repertoire variability, our study utilized correlation and ridge regression analyses on a subset of 81 samples, each encompassing both cell proportion and TCRβ sequencing data.

### 2.11 Statistical Analysis

All subsequent statistical analyses were performed using R software (Version 4.1.2). For binary group comparisons, the nonparametric Wilcoxon test was applied, whereas the Kruskal–Wallis test was employed for multigroup comparisons. Multiple testing adjustments were conducted using the false discovery rate (FDR) approach to maintain the integrity of statistical significance.

For all correlation analyses in this study, we primarily conducted Spearman rank correlation, Pearson correlation and linear regression analyses. To calculate the confidence intervals for Spearman correlation coefficients, we employed the Bootstrap method. This involved repeatedly resampling our original dataset 1000 times, computing the Spearman correlation coefficient for each resampled set. We then derived the 95% confidence intervals from these bootstrapped coefficients using the percentile method.

## 3 Results

### 3.1 Delineation of Peripheral Blood TCRβ Repertoire Reference Values in a Healthy Pediatric Cohort

**FIGURE 1:**
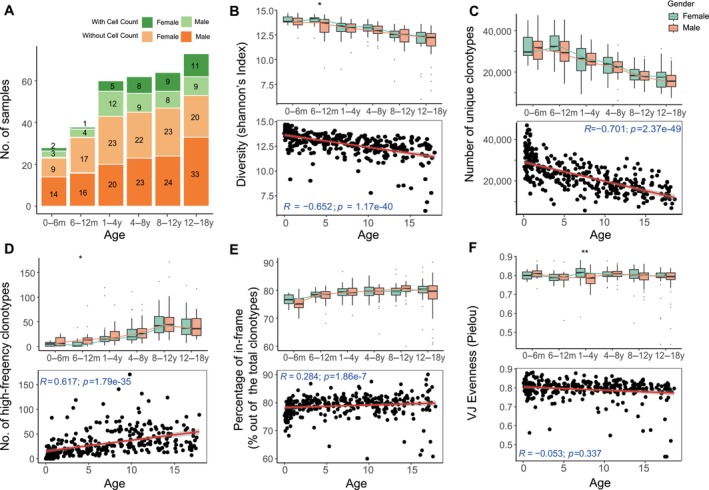
Age‐based grouping and TCRβ diversity indices in healthy children. (A) Sample size and gender information for each age group. (B) Age‐correlation analysis of the Shannon index indicating TCRβ diversity. (C) Age‐correlation analysis of unique clonotype number. (D) Trend of high‐frequency clonotype number with increasing age. (E) Changes in the proportion of functional clonotypes over time, specifically examining how the percentage of in‐frame clonotypes compares to total clonotypes with aging. (F) Age‐related variation in the CR4 index reflecting VJ gene usage. Spearman correlation coefficients and corresponding *p*‐values are indicated for (B–F). The asterisks above each group indicate the results of significance tests between males and females. Asterisks represent p‐values (*p ≤ 0.05; **p ≤ 0.01), with multiple testing corrections applied.

High‐throughput TCRβ IR sequencing was conducted on peripheral blood samples from 346 healthy children, ranging in age from 0 to 18 years. After discarding samples for various technical reasons, 11 samples due to total clonotype counts below 1million (1×10^6^) and 10 samples lacking necessary phenotypic information, a cohort of 325 samples was retained. This cohort consisted of 175 males and 150 females. Leveraging established reference values for peripheral blood lymphocyte subsets in children (Ding et al. 2018), we categorized the subjects into six distinct age brackets: 0–6 months, 6–12 months, 1–4 years, 4–8 years, 8–12 years, and 12–18 years. Distribution of sample sizes and gender across these age groups is depicted in Figure 1A.

Our study concentrated on examining the clonal dynamics of the TCRβ IR in healthy children, with a focus on how these dynamics vary with age and gender. We performed a thorough quantitative analysis encompassing parameters such as diversity, unique clonotype abundance, dominant clonotype frequency, the balance of in‐frame and out‐of‐frame clonotypes, and V/J gene usage evenness, as quantified by Pielou's index. The resulting age and gender‐specific distributions of these TCRβ repertoire characteristics are meticulously summarized in Table 1. This comprehensive dataset not only illuminates the immunological variability across different developmental stages but also provides a fundamental reference for future pediatric immunological research, establishing a benchmark in the field.

**TABLE 1:** Reference values of peripheral blood TCRβ repertoire characteristics in healthy children.

Metrics	Gender	Age					
		0–6 m	6–12 m	1–4 y	4–8 y	8–12 y	12–18 y
Unique clonotype	F	28,514 (24,443–42,314)^a^	31,149 (24,258–37,938)	25,410.5 (16,730–32,887)	22,786 (16,449–28,666)	17,266 (13,553–23,775)	16,586 (10,425–25,028)
	M	30,502 (21,074–37,996)	28,183.5 (18,551–41,267)	23,919 (21,114–29,926)	21,389.5 (15,514–27,667)	16,808 (12,762–22,606)	14,560 (10,757–22,029)
Shannon's H (TCR)	F	13.72 (13.495–14.39)	14.001 (13.461–14.32)	13.263 (11.713–13.687)	13.119 (12.081–13.612)	12.423 (11.795–13.078)	12.256 (11.188–13.206)
	M	13.695 (13.154–14.368)	13.579 (11.936–14.417)	13.083 (12.372–13.751)	12.79 (11.914–13.599)	12.404 (10.952–12.994)	12.108 (10.437–12.998)
Pielou's evenness (TCR)	F	0.928 (0.925–0.945)	0.931 (0.915–0.949)	0.906 (0.849–0.93)	0.906 (0.86–0.926)	0.886 (0.847–0.914)	0.871 (0.813–0.913)
	M	0.934 (0.894–0.94)	0.917 (0.816–0.941)	0.898 (0.845–0.927)	0.894 (0.853–0.92)	0.878 (0.797–0.912)	0.876 (0.772–0.909)
Gini skewing index (TCR)	F	0.595 (0.547–0.606)	0.58 (0.538–0.622)	0.622 (0.577–0.662)	0.635 (0.594–0.673)	0.662 (0.628–0.702)	0.665 (0.617–0.718)
	M	0.588 (0.55–0.642)	0.611 (0.551–0.68)	0.635 (0.593–0.695)	0.64 (0.597–0.681)	0.681 (0.63–0.744)	0.664 (0.615–0.749)
Clonotype CR4	F	0.507 (0.259–1.182)	0.419 (0.283–5.044)	3.883 (0.942–14.727)	3.103 (1.02–9.417)	4.757 (1.327–11.546)	6.732 (1.871–16.663)
	M	0.723 (0.357–6.962)	1.613 (0.457–11.571)	3.327 (0.998–10.354)	4.871 (0.938–13.014)	5.14 (1.822–15.862)	7.731 (2.747–21.197)
Top 100 clonotype (% of total clonotypes)	F	6.237 (3.661–7.362)	5.875 (3.783–10.225)	11.591 (6.916–22.998)	12.435 (7.21–22.723)	17.48 (9.836–24.004)	19.857 (8.785–29.343)
	M	6.436 (4.617–14.257)	8.456 (4.59–27.754)	12.187 (7.511–21.379)	14.804 (8.71–24.559)	17.642 (10.659–31.438)	19.988 (11.583–39.978)
No. of high‐frequency clonotype	F	5 (0–10)	1.5 (0–14.6)	15.5 (7.4–35.6)	19 (9–58.7)	42 (19.4–74.8)	37 (13–64)
	M	6 (0–23.8)	13.5 (1.8–26.5)	16.5 (6–39.8)	26 (10.3–60.4)	44 (23.1–82.2)	36 (19.1–70.7)
Shannon's H (VJ)	F	7.493 (7.279–7.743)	7.382 (6.965–7.709)	7.625 (7.21–7.968)	7.516 (7.184–7.812)	7.512 (7.171–7.876)	7.425 (6.294–7.713)
	M	7.542 (7.366–7.95)	7.406 (7.132–7.622)	7.372 (6.889–7.73)	7.569 (7.244–7.812)	7.479 (6.76–7.66)	7.421 (6.69–7.673)
Gini skewing index (VJ)	F	0.762 (0.746–0.787)	0.777 (0.74–0.803)	0.741 (0.684–0.793)	0.753 (0.708–0.786)	0.752 (0.713–0.787)	0.764 (0.729–0.845)
	M	0.754 (0.697–0.773)	0.772 (0.75–0.803)	0.772 (0.73–0.809)	0.753 (0.722–0.781)	0.759 (0.737–0.811)	0.76 (0.731–0.813)
Pielou's evenness (VJ)	F	0.8 (0.78–0.825)	0.79 (0.748–0.827)	0.815 (0.772–0.857)	0.804 (0.77–0.838)	0.803 (0.771–0.844)	0.796 (0.68–0.825)
	M	0.81 (0.786–0.851)	0.792 (0.763–0.813)	0.787 (0.738–0.825)	0.81 (0.776–0.835)	0.802 (0.722–0.822)	0.795 (0.716–0.821)
VJ CR4	F	13.269 (10.871–15.617)	15.691 (11.273–21.838)	13.491 (9.636–20.75)	14.228 (10.462–19.553)	15.604 (9.819–19.764)	15.981 (12.854–28.832)
	M	13.191 (7.853–17.172)	15.397 (12.069–18.973)	16.361 (10.914–26.089)	15.205 (10.054–18.284)	16.336 (12.082–27.575)	16.194 (12.548–31.157)
Frequency of in‐frame clonotypes (% of total clonotypes)	F	76.69 (73.31–78.78)	78.45 (76.444–80.291)	79.39 (77.224–81.642)	79.65 (76.262–81.953)	79.665 (76.67–82.339)	80.39 (76.86–83.92)
	M	75.13 (72.11–79.4)	78.615 (74.162–80.887)	79.265 (76.824–82.119)	79.715 (75.576–81.444)	80.58 (76.564–82.799)	79.72 (70.29–83.921)
Frequency of nonfunctional clonotypes (% of total clonotypes)	F	0.48 (0.38–0.77)	0.52 (0.337–1.839)	0.52 (0.311–1.328)	0.525 (0.239–1.142)	0.515 (0.256–1.537)	0.42 (0.21–0.81)
	M	0.64 (0.356–2.588)	0.44 (0.27–0.78)	0.51 (0.256–0.71)	0.48 (0.33–1.63)	0.39 (0.27–0.588)	0.46 (0.291–1.604)
Frequency of out‐of‐frame clonotypes (% of total clonotypes)	F	22.99 (20.28–26.17)	20.5 (19.323–22.377)	19.965 (17.616–22.201)	19.495 (16.949–23.023)	19.665 (17.142–22.157)	19.47 (15.56–22.83)
	M	24.34 (20.154–25.89)	20.855 (18.825–25.339)	20.24 (17.431–22.224)	19.695 (17.783–23.314)	18.85 (16.949–22.822)	19.295 (15.565–29.275)
Frequency of out‐of‐frame (CDR3_length) clonotypes (% of total clonotypes)	F	18.88 (16.48–22.23)	16.51 (15.163–18.44)	15.87 (13.829–18.203)	15.415 (13.163–18.175)	15.49 (12.908–17.951)	14.99 (11.3–18.21)
	M	20.17 (16.76–21.698)	16.94 (14.489–21.939)	16.195 (13.116–17.888)	15.67 (14.311–18.806)	15.205 (13.156–18.985)	15.185 (11.49–26.097)
Frequency of out‐of‐frame (Stop_Codon) clonotypes (% of total clonotypes)	F	3.94 (3.36–4.26)	3.98 (3.298–4.25)	4.095 (3.115–4.431)	4.07 (3.685–4.897)	3.98 (3.275–4.735)	4.27 (3.71–4.74)
	M	3.82 (3.446–4.388)	4.03 (3.664–4.311)	4.11 (3.711–4.429)	3.9 (3.371–4.438)	3.79 (3.302–4.443)	4.145 (3.303–4.891)

^a^ The numbers outside the parentheses represent median values, whereas those inside detail the 10th to 90th percentile range.

Unique clonotype: The number of distinct TCR clonotypes present in the repertoire. Each clonotype represents a unique CDR3 amino acid sequence. Shannon's H (TCR): Shannon diversity index, a measure of the diversity within the TCR repertoire. Higher values indicate greater diversity of TCR sequences. Pielou's evenness (TCR): Pielou's evenness index measures the evenness of the distribution in relation to the total diversity. Values close to 1 indicate a very even distribution. Gini skewing index (TCR): Gini coefficient focuses on the inequality or skewness of the abundance distribution. Higher values indicate a greater dominance of certain clonotypes, suggesting a more uneven distribution. Clonotype CR4: The cumulative frequency of the top 4 most abundant clonotypes in the TCR repertoire, indicating the concentration of dominant clonotypes. Top 100 clonotype (% of total clonotypes): The percentage of the top 100 most abundant clonotypes in the total number of clonotypes, reflecting the proportion of dominant clonotypes in the TCR repertoire. No. of high‐frequency clonotype: The number of high‐abundance clonotypes, typically referring to clonotypes with higher frequencies (≥ 0.1%) within the TCR repertoire. Shannon's H (VJ): Shannon diversity index for the diversity of VJ recombination. Gini skewing index (VJ): Gini coefficient for the abundance distribution inequality of VJ recombination. Pielou's evenness (VJ): Pielou's evenness index for the evenness of VJ recombination. VJ CR4: The cumulative frequency of the top 4 most abundant VJ combinations. Frequency of in‐frame clonotypes (% of total clonotypes): The frequency of TCR sequences that are in‐frame recombinations, which can translate into functional TCR proteins. Frequency of nonfunction clonotypes (% of total clonotypes): The frequency of TCR sequences with variable (V) and joining (J) genes classified as pseudogenes in the IMGT database, rendering the TCRs nonfunctional. Frequency of out‐of‐frame clonotypes (% of total clonotypes): The frequency of TCR sequences that are out‐of‐frame recombinations, including two types: out‐of‐frame (CDR3_length) and out‐of‐frame (Stop_Codon). Frequency of out‐of‐frame (CDR3_length) clonotypes (% of total clonotypes): The percentage of TCR sequences that are out‐of‐frame due to abnormal lengths in the CDR3 region, where the TCR sequence length is not a multiple of three. Frequency of out‐of‐frame (Stop_Codon) clonotypes (% of total clonotypes): The frequency of out‐of‐frame TCR sequences due to the presence of premature stop codons.

### 3.2 Age‐Related Changes in TCRβ Repertoire Diversity and Clonality in Childhood Development

In our detailed analysis of TCRβ diversity and clonality throughout childhood development, we employed Spearman's correlation to investigate the relationship between age and various metrics of the TCRβ repertoire (Figure 1B–F).

We observed a notable decrease in both the Shannon index and the unique clonotype number—primary indicators of TCRβ diversity—as age increased. This pattern, indicating reduced TCRβ diversity with advancing age, was supported by R values of −0.65 (95% confidence interval [CI]: [−0.72, −0.57]) and −0.70 (95% CI: [−0.76, −0.64]) for the Shannon index and unique clonotype number, respectively, with statistical significance (*p* < 0.001; Figure 1B,C). These results align with previous research in adult populations showing a decline in TCRβ diversity with age (Yager et al. 2008; Sun et al. 2022). Additionally, similar trends of diminishing TCRβ diversity and increased clonality have been observed across a wider age range (0–89 years) in a cohort of 821 allergy patients (Cao et al. 2020).

Conversely, our data revealed an increase in high‐frequency clonotypes and percentage of in‐frame clonotypes with age. The number of high‐frequency clonotypes showed a positive correlation with age (*R* = 0.62, *p* < 0.001, 95% CI: [0.53, 0.69]; Figure 1D), whereas the percentage of in‐frame clonotypes exhibited a slight positive trend (*R* = 0.28, *p* < 0.001, 95% CI: [0.17, 0.40]; Figure 1E). These findings suggest a complex maturation of the TCRβ repertoire in healthy children, warranting further exploration. Additionally, the Pielou index, indicative of V/J gene usage uniformity, demonstrated a nonsignificant negative correlation with age (*R* = −0.05, *p* = 0.34, 95% CI: [−0.16, 0.05]; Figure 1F).

To validate our findings, we analyzed an independent dataset comprising 25 healthy individuals aged 0–20 years. Analysis of key TCR repertoire metrics revealed remarkably consistent age‐associated patterns with our primary cohort (Figure S1). Specifically, we observed that diversity metrics (Shannon diversity index and number of unique clonotypes) showed significant negative correlations with increasing age, whereas clonality metrics (number of high‐frequency clonotypes, percentage of in‐frame clonotypes, percentage of top 100 clonotypes, and Gini coefficient) showed significant positive correlations with age. These striking parallels between our cohort and the validation dataset reinforce the robustness of our observations and suggest conserved developmental patterns in immune system maturation across populations.

Further analysis revealed no significant gender‐based differences in these TCR repertoire metrics, both in our primary cohort across different age groups (Figure S2A) and in the validation dataset (Figure S2B).

Overall, our results highlight that age substantially influences the diversity and clonality of the TCRβ repertoire, whereas gender seems to have a lesser impact. The observed variations in metrics, such as the Shannon index and high‐frequency clonotypes across different ages, reinforce the existing knowledge on age‐related changes in the IR.

### 3.3 Dynamic Changes in CDR3 Length and V(D)J Gene Usage Implicate Age‐Related Immune Diversity

**FIGURE 2:**
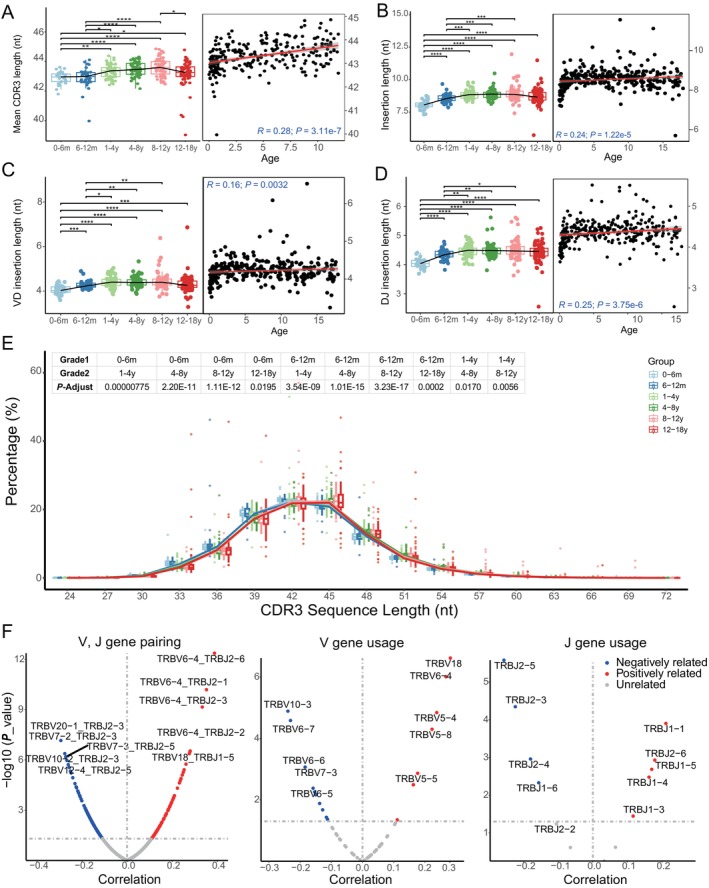
Age‐associated variations in CDR3 length and VDJ gene dynamics in healthy individuals. (A) Progressive increase in the average CDR3 length from ages 0 to 12, followed by a shortening trend post 12 years. (B) General upward trend of insertion lengths with advancing age. (C) Correlation of VD insertions with age, showing a positive, albeit modestly significant, relationship. (D) Relationship between DJ insertions and age, illustrating a positive correlation. (E) Distribution of CDR3 lengths across different age groups, highlighting an enrichment of sequences with longer CDR3 regions as age increases. The upper matrix displays pairwise comparisons between groups, where only statistically significant differences are shown (adjusted *p* < 0.05). (F) Dynamic correlation of VJ gene pairings and VJ gene usage with age. Annotations indicate the Pearson correlation coefficients, with corresponding *p*‐values indicated. Spearman correlation coefficients and corresponding *P*‐values are indicated for (A–D). The asterisks above each group indicate the results of significance tests between each pair of groups. Asterisks above the lines represent *p*‐values between the two groups at the ends of the line (*p ≤ 0.05; **p ≤ 0.01; ***p ≤ 0.001; ****p ≤ 0.0001), with false discovery rate control applied.

The length and composition of the CDR3 region are crucial for adaptive immune responses to a wide range of antigens and may increase the risk of autoimmune diseases by enhancing self‐antigen recognition (Gomez‐Tourino et al. 2017; Liu et al. 2019). Our investigation into the CDR3 region and V(D)J gene usage across different ages revealed significant patterns. An increase in the average CDR3 length from birth to 12 years (*R* = 0.42, *p* < 0.001, 95% CI: [0.31, 0.52]; Figure 2A) suggests a developmental elongation, potentially enhancing immune responses to environmental antigens. However, a trend of declining CDR3 length is observed post 12 years (Figure 2A, Bonferroni‐corrected *p*‐values). It is worth noting that outliers may influence these results. Future studies with larger sample sizes could help robustly detect and characterize these potential age‐related changes in CDR3 length.

To understand the age‐related decline in clonal diversity, we specifically analyzed V(D)J gene usage and the impact of insertions and deletions within these genes on clonal diversity. Our analysis showed consistent deletions in V3, D5, D3, and J5 genes across all age groups (Figure S3). However, insertion events presented a more complex pattern. We observed a general trend of increasing insertion lengths with age (*R* = 0.24, *p*‐value < 0.001, 95% CI: [0.12, 0.35]; Figure 2B). The correlation between age and VD insertions showed a modest but statistically significant trend (*R* = 0.16, *p* = 0.0032, 95% CI: [0.05, 0.28]; Figure 2C). In contrast, the relationship between DJ insertions and age was more evident, exhibiting strong statistical significance (*R* = 0.25, *p* < 0.001, 95% CI: [0.14, 0.36]; Figure 2D).

Furthermore, our analysis highlighted significant variations in CDR3 length distributions across different age groups. Notably, as individuals aged, there was a prominent increase in the frequency of sequences with longer CDR3 regions (Figure 2E).

In examining the abundance and pairing patterns of V and J genes, we observed distinct age‐related trends. V genes, such as *TRBV6‐6* and *TRBV18*, and J genes, such as *TRBJ2‐6* and *TRBJ1‐5*, showed increased utilization with advancing age. Conversely, the use of genes such as *TRBV6‐5*, *TRBV7‐3*, and *TRBJ2‐5* saw a gradual decrease over time (Figure 2F).

Collectively, the observed age‐related changes in clonal diversity can be largely attributed to the evolving patterns of insertions within V(D)J genes. This study delineates a clear distinction in the behaviors of VD and DJ insertions, with DJ insertions showing a more pronounced correlation with age. Additionally, variations in CDR3 length distribution and shifting preferences in TCR gene usage across different age groups further emphasize the dynamic changes in the adaptive immune system with age.

### 3.4 Dynamic Clonal Changes Might Reflect Perinatal Imprints and the Progressive Personalization of the Immune System Into Adulthood

**FIGURE 3:**
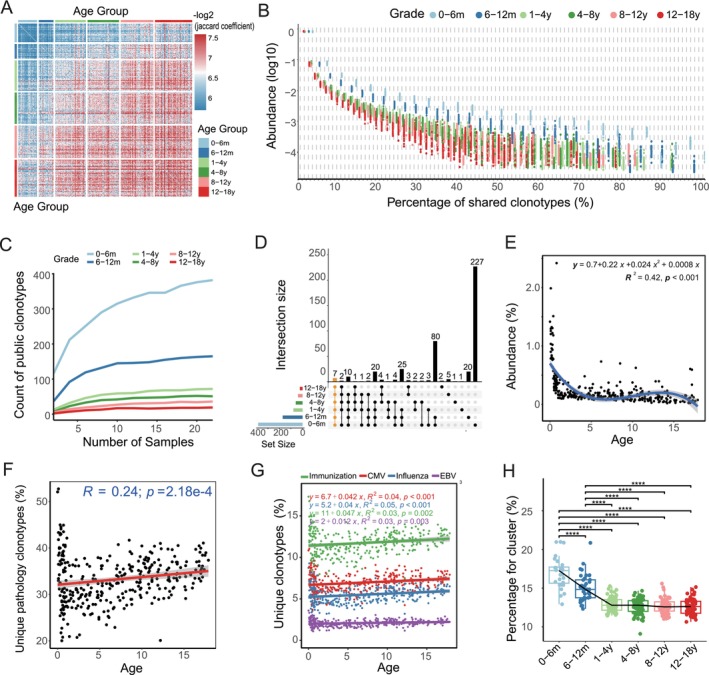
Age‐related dynamics in TCRβ repertoire diversity and pathogen‐related clonotype annotations. (A) Similarity of TCRβ clonotypes across samples, quantified by ‒log2‐transformed Jaccard indices. (B) Trend of shared clonotype abundance in different age groups as a function of the proportion of samples sharing the clonotype (*x*‐axis represents the proportion of samples sharing the clonotype). (C) Variation in the number of public clonotypes (defined as clonotypes shared by more than 50% of samples) across different age groups with increasing sample size. (D) Comparative analysis of public clonotypes across different age cohorts. The dots represent individual data points for each age group. The lines are used to help visually trace the data across the groups. (E) Negative correlation between the abundance of public clonotypes in the corresponding age subgroups and the age of the samples. (F) Spearman correlation between the abundance of public clonotypes and sample age, with public clonotypes specific to the respective age cohort. (G) Correlation between the percentage of unique clonotypes related to exogenous microbial infections and sample age. (H) Variations in the proportions (defined by the ratio of clusters identified by GLIPH2 to unique clonotypes) of clusters among different age groups using GLIPH2.

Previous studies have suggested that TCRs shared among different individuals may reflect disease‐specific clonotypes (Gomez‐Tourino et al. 2017; Liu et al. 2019) or common pathogen exposures (Sun et al. 2022). On the basis of this, our study delved into the age‐related dynamics of TCRβ clonotypes in pediatric populations. The heatmap analysis, using the Jaccard coefficient, indicated a significant decrease in TCRβ repertoire similarity with age (Figure 3A), pointing toward evolving immune responses or exposures over time.

Further, we observed dynamic changes in the abundance and shared percentage of TCRβ clonotypes across pediatric age groups (Figure 3B). The youngest cohort (0–6 months) showed a high abundance of shared clonotypes, possibly indicating uniform early life antigen exposure. However, as age increased, a decline in clonotype abundance was noted, suggesting a transition to a more diverse and individualized immune memory.

To understand the influence of sample size on public clonotype counts (clonotypes shared by more than 50% of samples), we analyzed data across different age groups (Figure 3C). The analysis revealed that the number of public clonotypes increases with sample size but plateaus around 10 samples. Notably, in the youngest group (0–6 months), there is a rapid accumulation of public clonotypes, even with fewer samples. In contrast, older groups, especially the 12–18 years cohort, exhibit a low and stable number of public clonotypes regardless of sample size, indicative of a shift toward a more diversified and personalized immune response with age.

Further comparative analysis showed that public clonotypes are most abundant in the 0–6 months cohort, with a notable overlap observed with other age groups (Figure 3D). To delve deeper into the age‐specific distribution of these clonotypes, we examined the presence of public clonotypes unique to each age subgroup and those shared across two or more subgroups (Figures S3 and S4). The data showed a pronounced presence of public clonotypes within the youngest cohort, with a declining trend observed as age increased. Moreover, analysis indicated a significant negative correlation between the abundance of public clonotypes and the age of the subgroups (*R* ^2^ = 0.42, *p* < 0.001, Figure 3E), highlighting a maturation and diversification of the IR with advancing age.

To evaluate whether the public clonotypes identified in our cohort are population specific or universally distributed, we analyzed their reproducibility in an independent dataset of 25 healthy individuals aged 0–20 years. Remarkably, we found that the public clonotypes identified across different age groups in our cohort showed high reproducibility (reproducibility rate > 94%) in the validation dataset (Figure S5A). This high degree of conservation suggests that public clonotypes identified during early life development are shared across different populations, potentially serving similar functional roles during this critical developmental period.

The observed pattern, particularly the high abundance of shared clonotypes in early life transitioning to a more varied profile in older children, aligns with existing research. It supports the concept that clonal expansions in the perinatal period have a lasting impact on the T‐cell repertoire, which is then gradually reshaped by diverse clonal selection into adulthood, leading to a more varied and individualized immune system (Gaimann et al. 2020).

### 3.5 Early Life Vaccinations and Antigenic Exposures May Establish Foundations for Lifelong Immune Personalization

In our endeavor to understand the characteristics of public clonotypes, we first utilized cluster analysis with the Levenshtein distance (Figure S5B). This revealed a notable tendency for clustering among public clonotypes in younger age groups, suggesting that these clonotypes may share distinct immune responses or common antigenic exposures. Intriguingly, we found that these public clonotypes often had shorter CDR3 regions compared to randomly selected clonotypes within the same age cohorts (Figure S5C). This correlation between clustering and shorter CDR3 lengths in younger age groups likely reflects the limited V(D)J combinatorial diversity and junctional diversity in early life. The shorter CDR3 regions may represent a more germline‐like state due to reduced nontemplated nucleotide insertion/deletion, indicating limited clonal refinement before extensive antigen exposure and selection.

Furthermore, we expanded our analysis by annotating public clonotypes with known pathology‐associated clonotypes. The results indicated an age‐dependent increase in TCR clonotypes associated with pathogenic infections (*R* = 0.20, *p* < 0.001, 95% CI: [0.09, 0.33]; Figure 3F), particularly those targeted by common vaccinations and infections such as CMV, influenza virus, and EBV‐related TCR clonotypes (Figure 3G). This suggests that as children age, their TCR repertoire evolves in response to an expanding range of pathogenic exposures.

Additionally, our GLIPH2 analysis for unique clonotype clustering showed that clusters were significantly more prevalent in the 0–1‐year age group, especially within the 0–6‐month bracket (Figure 3H), suggesting greater diversity in the TCR repertoire during early life.

These observations suggest a potential role for early life antigenic exposures, including those vaccinations and exposure to common viruses, in shaping the TCRβ repertoire. The lower incidence of pathology‐associated clonotypes in infants (Figure 3G) aligns with limited antigen exposure, whereas the elevated repertoire diversity and lower proportion of in‐frame clonotypes at this stage (Figure 1E) highlight the developing nature of the immune system. With increasing age and antigen exposure, there is a corresponding rise in both in‐frame and pathology‐associated clonotypes (Figures 1E and 3G), supporting the notion that antigen exposure influences the selection and expansion of functional TCR clones.

It is important to note that although our participants followed China's National Immunization Program, the absence of individual vaccination records prevents direct correlation of vaccinations with repertoire diversity. Thus, although the age‐related increase in pathology‐associated clonotypes likely reflects TCR repertoire maturation influenced by vaccines, natural infections, and environmental factors. Further studies with detailed immunization histories are required to delineate the specific effects of vaccinations on TCRβ diversity and specificity.

### 3.6 TCRβ Repertoire Characteristics Possess Potential for Predicting Biological Age

**FIGURE 4:**
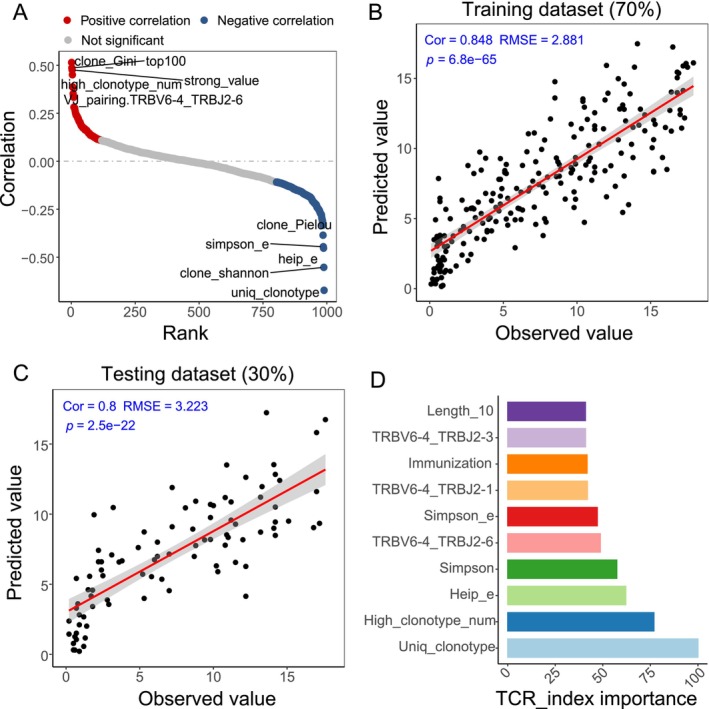
Construction of the immune age model based on TCRβ repertoire characteristics. (A) Correlation between TCRβ repertoire features and chronological age (Pearson correlation coefficient). Red dots indicate significant positive correlation, blue dots indicate significant negative correlation, and gray dots denote no significant correlation. (B) Predicting biological age of samples (from 70% training set) using TCRβ repertoire features that show significant correlation with age. A principal component regression method was employed for model construction, highlighting the correlation between predicted immune age and the actual biological age of samples. (C) The validity of the immune age prediction model is further assessed in a validation set (comprising 30% of the samples). (D) Ranking of feature importance in the immune age prediction model (Top 10). Length_10: Represents the frequency of CDR3 regions with a length of 10 amino acids. TRBV6‐4_TRBJ2‐3, TRBV6‐4_TRBJ2‐1, and TRBV6‐4_TRBJ2‐6: Indicates the frequency of specific TCR beta variable (TRBV) and joining (TRBJ) gene combinations within the TCR repertoire, reflecting the diversity of TCR combinations. *Immunization*: Refers to the percentage of clonotypes that are amplified in response to vaccination. *Simpson_e*: Measures Simpson's evenness index, which quantifies the diversity of TCR clonotypes focusing on the equal representation of various clonotypes. *Heip_e*: Measures the evenness of clonal distribution, sensitive to variations in clonotype abundance, especially useful in identifying diversity within rare clonotypes. *High_clonotype_num*: The number of high‐abundance clonotypes, typically referring to clonotypes with higher frequencies (≥ 0.1%) within the TCR repertoire. *Uniq_clonotype*: Counts the distinct TCR clonotypes based on unique CDR3 amino acid sequences.

To investigate the potential of the TCRβ repertoire as a biomarker for biological age, we initially explored the association between various TCRβ repertoire characteristics and biological age. Our analysis revealed differential associations with age: high‐frequency clonotype number and the Gini index of clonotypes displayed a positive correlation with age, whereas metrics such as the Shannon index, Pielou index, and the count of unique clonotypes were inversely related to age (Figure 4A).

Extending our findings, we delved into predicting biological age using TCRβ repertoire characteristics. Utilizing 70% of the dataset for model training, we employed a principal component regression model. The model demonstrated significant predictive power, as evidenced by a strong correlation between predicted and actual biological ages (correlation coefficient (Cor) = 0.848, root mean square error (RMSE) = 2.881, *p* = 6.8e‐65, 95% CI: [0.81, 0.88]; Figure 4B). To validate the model's efficacy, we applied it to an independent dataset, constituting the remaining 30% of the samples. This validation process reaffirmed the model's consistency and accuracy (Cor = 0.8, RMSE = 3.223, *p* = 2.5e‐22, 95% CI: [0.71, 0.86]; Figure 4C).

Further analysis refined our understanding of the key TCRβ features contributing to age prediction. Among these, the count of unique clones, the number of high‐frequency clonotypes, and attributes related to immune response emerged as particularly influential (Figure 4D). These critical features not only enhance our model's predictive capability but also provide insights into the immune system's evolution across different age groups.

### 3.7 Age‐Dependent Shifts in T‐Cell Subpopulations and Their Potential Effects on TCRβ Repertoire Diversity

**FIGURE 5:**
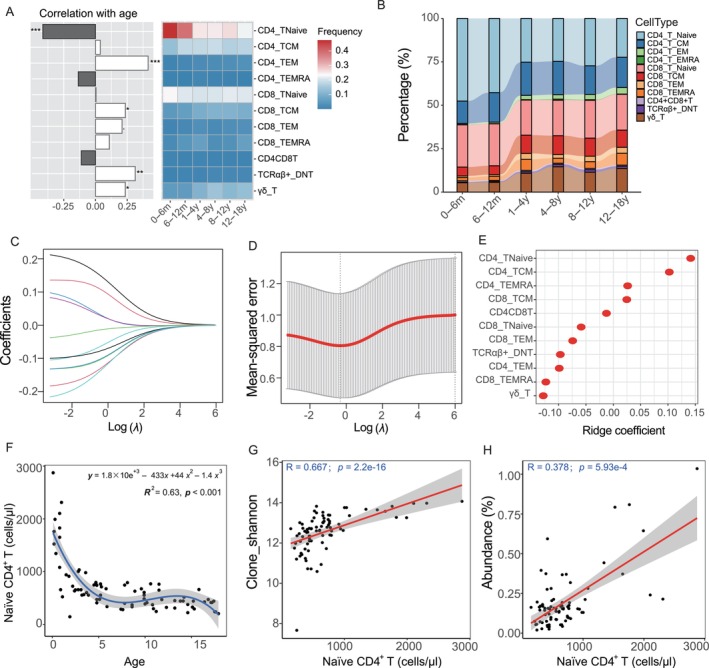
Analysis of T‐cell subgroup proportions in 81 healthy Chinese children across different ages. (A) Age‐associated variations in CD4 Tem, CD8 T‐cells, and NK cell subgroup percentages. Asterisks indicate the significance of correlations between cell subtypes and age (*p ≤ 0.05; **p ≤ 0.01; ***p ≤ 0.001), with false discovery rate control applied. (B) Depicting the progressive reduction in proportions of CD4 T naïve and CD8 T naïve with increasing biological age. (C) Ridge regression coefficients for various independent variables at different log (*λ*) values. (D) Ten‐fold cross‐validation results highlighting an optimal log(lambda) value slightly below zero, corresponding to the minimal mean‐squared error. (E) Ranking of ridge regression coefficients for T‐cell subgroups, emphasizing the predictive significance of CD4 T naïve. (F) Detailed age‐based trend for CD4 T naïve cells, marking a decline (0–5 years), stabilization (5–14 years), and subsequent drop (> 15 years). (G, H) Positive correlations between the count of CD4 T naïve cell and both the Shannon index (*R* = 0.67, *p* = 2.2e‐16) and the abundance of public clones (*R* = 0.38, *p* = 5.93e‐4), underlining the role of CD4 T naïve in T‐cell repertoire diversity. Both correlations were assessed using the Spearman's rank correlation test.

In our previous investigations, we established the baseline distributions of peripheral blood lymphocyte subgroups in a healthy Chinese pediatric cohort, ranging from newborns to 18‐year‐olds. This foundational study highlighted distinct age‐associated variations in CD4 and CD8 T‐cell subpopulations, both in terms of percentages and absolute counts (Ding et al. 2018). Building on this, we conducted a comprehensive analysis of 81 samples from this cohort, focusing on cell proportion data. Our findings indicate significant age‐related increases in the proportions of CD4 Tem, CD8 Tcm, TCRαβ^+^ DNT, and γδ T‐cell, alongside a notable decrease in CD4 T naïve cells (Figure 5A,B). These findings within our pediatric cohort are in line with prior research on young and aged populations (Li et al. 2019; Zheng et al. 2020), indicating consistent patterns of immune system remodeling from early childhood to advanced age.

To elucidate the impact of T‐cell subpopulation dynamics on the diversity of TCRβ repertoire, we applied ridge regression analysis. Our findings, depicted in Figure 5C, show that the regression coefficients for different T‐cell subgroups exhibited a trend toward zero as the log lambda values increased, indicating that our model becomes more conservative with higher levels of regularization. This approach results in more consistent estimations of T‐cell proportions across different age groups. Through 10‐fold cross‐validation, we identified an optimal lambda value with a slight negative bias that minimized the mean‐squared error (Figure 5D). Further scrutiny of the model identified the CD4 T naïve subgroup as having the highest predictive coefficient, followed by CD4 Tcm, CD4 Temra, and CD8 Tcm (Figure 5E), underlining their relative importance in predicting changes in TCRβ repertoire diversity.

Remarkably, we noted a significant decrease in the count of CD4 T naïve cells between ages 0–5 years, a stable phase from 5 to 14 years, and a substantial reduction post 15 years (Figure 5F). This pattern mirrored the trend in public clonotype abundance over these age ranges. Stimulated by this finding, we investigated the correlation between the count of CD4 T naïve cell and factors such as clonal diversity and public clonotype abundance. The analysis showed a positive correlation of the count of CD4 T naïve with the Shannon index (*R* = 0.67, *p* = 2.2e‐16, 95% CI: [0.52, 0.77]; Figure 5G) and the abundance of public clones (*R* = 0.38, *p* = 5.93e‐4, 95% CI: [0.14, 0.57]; Figure 5H), suggesting that the observed decline in the TCRβ repertoire's diversity and reduced public clonotype prevalence are largely attributable to the decreasing count of CD4 T naïve cells in T‐cells.

## 4 Discussion

TCR repertoire diversity is crucial for understanding adaptive immune system development, particularly in pediatric populations. Our study utilized multiplex PCR and high‐throughput sequencing to examine the age‐related dynamics and spectral characteristics of TCRβ repertoire in 325 Chinese children spanning age 0–18 years, providing valuable insights into the evolving immune profiles during critical stages of immunological development.

Our study revealed decreased TCRβ repertoire diversity and increased clonality with age. These findings align with previous studies that have examined broader age ranges (Britanova et al. 2016, 2014; Yoshida et al. 2017; Gil et al. 2015; Pogorelyy et al. 2017), suggesting that these changes are part of a continuous process throughout the human lifespan. For instance, similar age‐related shifts in the TCRβ repertoire have been reported in studies of individuals aged 6–90 years (Britanova et al. 2014) and in a 20‐year longitudinal examination of adults aged 23–65 years (Yoshida et al. 2017). Importantly, our observation that TCRβ repertoire diversity varies with age rather than gender is corroborated by a study of 154 Chinese individuals aged 6–70 years (Lian et al. 2019), underscoring the importance of using age‐matched controls in future research.

The reduction in TCRβ repertoire diversity and increased clonality with age likely stem from two primary factors. First, we observed an age‐related increase in the CDR3 region length, particularly due to the elongation of insertion lengths contributing to junctional diversity. Second, we noted shifts in V and J gene usage patterns, with certain genes (e.g., *TRBV6‐6* and *TRBV18*), becoming more dominant with age, whereas others such as *TRBV6‐5* and *TRBV7‐3* tend to decline. These alterations in CDR3 length and gene usage patterns collectively contribute to the observed changes in TCRβ repertoire composition over time.

Further analysis revealed a notable decline in TCRβ clonotype similarity as children age, with significant clonotype clustering in the 0–1‐year age group, particularly within the first 6 months. We found that public clonotypes, which were abundant in younger age groups, exhibited shorter CDR3 regions and decreased in prevalence over time. This pattern aligns with research suggesting that T‐cell clones generated during prenatal development persist long term (Gaimann et al. 2020; Pogorelyy et al. 2017; Khosravi‐Maharlooei et al. 2019; Smith et al. 2021; Zvyagin et al. 2014), as demonstrated by studies showing similar TCR recombination patterns between related and unrelated individuals (Putintseva et al. 2013). During prenatal development, reduced random nucleotide insertion leads to large, low‐diversity public clones that persist into adulthood (Pogorelyy et al. 2017), and these perinatal clonal expansions leave a lasting imprint on the TCR repertoire (Gaimann et al. 2020). Although maternal factors have been suggested to influence early repertoire development, studies indicate that shared peptide patterns between newborns and their mothers are comparable to those between newborns and unrelated pregnant women (Guo et al. 2016), suggesting that developmental programming through thymic selection, rather than maternal inheritance, drives early life TCR repertoire formation.

The Levenshtein distance analysis further confirmed that public clonotypes have shorter CDR3 regions, aligning with existing research (Khosravi‐Maharlooei et al. 2019; Smith et al. 2021). These findings suggest that public CDR3β sequences are more similar to their germline origins, featuring shorter insertions and reduced CDR3 lengths (Khosravi‐Maharlooei et al. 2019). The persistence of these public clonotypes provides insights into the developmental trajectory of the TCR repertoire. Supporting our observations, studies comparing TCR repertoire overlaps between unrelated individuals and monozygotic twins (Zvyagin et al. 2014), as well as deep sequencing of T‐cell repertoires from diverse age groups and umbilical cord blood (Pogorelyy et al. 2017), indicate that many public clonotypes originate during fetal development and persist into adulthood, albeit with decreasing frequency over time. Furthermore, recent research on CD8^+^ T‐cells in older adults (van de Sandt et al. 2023) observed a transition from young public TCR clonotypes, typical in infants, to more diverse and private TCR clonotypes in older individuals, along with a reduction in young public motifs. This age‐related shift aligns with our findings on clonotype composition and diversity changes across different age groups.

Additionally, our analysis of the pathology‐associated clonotype annotation of public clonotypes across different age groups indicated an increase in pathogen‐related TCR clonotypes, particularly those linked to vaccinations and viral infections. This trend, coupled with an observed increase in the proportion of in‐frame clonotypes with age, suggests that the immune system develops a more functionally diverse repertoire over time, enhancing its ability to respond to a variety of antigens. These findings contribute to our understanding of the immune system's adaptability, especially during early life stages. The maturation process, influenced by environmental interactions and their effects on specific TCRs, highlights the immune system's plasticity across developmental stages. The observed impact of common pathogens and the formation of TCR clusters, potentially signaling collective immune exposures (DeWitt 3rd et al. 2018), underscore the complex interplay between environmental factors and immune development.

Furthermore, our study illuminates the potential of TCRβ repertoire characteristics as indicators of biological age. We found the number of unique clonotypes and diversity indices (Shannon and Pielou) negatively correlated with biological age, whereas high‐frequency clonotypes and the Gini index showed positive correlations. Utilizing these factors, we developed a principal component regression model for age prediction, which demonstrated significant accuracy in both training (70%) and independent (30%) validation datasets. The effectiveness of our model in predicting biological age based on TCRβ repertoire characteristics aligns with a study of 4066 Chinese adults, which reported an *R* ^2^ value of 0.37 for immune age prediction using TCR and BCR (Nie et al. 2022). These results collectively support the broad applicability and IR analysis across different age groups in assessing biological aging.

Finally, our analysis of 81 healthy pediatric samples revealed significant age‐related variations in CD4 and CD8 T‐cell subpopulations. Utilizing ridge regression analysis, we found that the proportions of T‐cell subgroups, including CD4 T naïve, CD4 Tcm, CD4 Temra, and CD8 Tcm, varied with age, influencing the diversity of the TCRβ repertoire. Notably, we observed a positive correlation between the proportion of CD4 T naïve cells and both clonal diversity and the percentage of unique clonotypes, suggesting a potential link between the decline in TCRβ repertoire diversity and reduction in CD4 T naïve cells with age.

These findings partially align with previous research on 30 adults (Sun et al. 2022), which reported a decrease in αβ TCR repertoire richness in both CD4^+^ and CD8^+^ T‐cells, particularly in naïve CD8^+^ Tcells, along with an increase in the clonal expansion of memory CD8^+^ T‐cells. However, our study reveals a more pronounced effect from CD4 T naïve cells. This difference in findings may be attributed to our focus on a different demographic, variations in sample size, study design, or analytical methods.

Importantly, large‐scale studies provide broader context for our findings. Analysis of immune characteristics in 1068 healthy adults demonstrated that the decline in CD4 naïve cells continues beyond age 18 (Qin et al. 2016), indicating that the IR remains dynamic throughout adulthood. Moreover, research in healthy individuals aged 20–65 has revealed that while CD4 naïve cell production decreases with age, their loss rate also declines as a compensatory mechanism (Mold et al. 2019). This suggests that the reduction in CD4 naïve cells is not solely attributable to their conversion to memory cells through antigen exposure but rather reflects a complex interplay of decreased thymic output and altered cell turnover rates.

The discrepancy between our findings and previous studies highlights the complex and potentially age‐dependent roles of different T‐cell subsets in shaping the TCR repertoire. Although both CD4^+^ and CD8^+^ T‐cells are known to play important roles in shaping the TCR repertoire (Li et al. 2016), our results suggest that the dynamics of CD4^+^ T naïve cells may have a more substantial impact on TCRβ repertoire diversity than previously thought, at least in our study population. Further research is needed to elucidate the specific mechanisms underlying these observed differences and their implications for immune system function across different age groups.

Although this study provides important insights into the diversity and characteristics of the TCRβ chain in a pediatric cohort, we acknowledge several limitations that point to future research opportunities. Our focus on the TCRβ chain has been informative; it presents an incomplete picture of the adaptive immune system. The absence of BCR analysis and limited exploration of the TCR α chain constrain our ability to fully comprehend the complex remodeling of immunity during aging. This limitation, coupled with our reliance on early collected DNA samples, underscores the need for more comprehensive approaches in future studies. Incorporating BCR analysis, deeper exploration of the TCR α chain, and utilizing advanced techniques like single‐cell RNA sequencing could provide a more holistic view of immune system dynamics.

The challenges we faced in defining ‘healthy’ in our pediatric cohort highlight a broader issue in pediatric immunology research. Despite our comprehensive screening processes, the dynamic nature of children's health means that undetected conditions may have been present or developed shortly after assessment. This emphasizes the need for longitudinal studies to capture the evolving nature of immune system development more accurately.

Furthermore, the specificity of our results to one population raises questions about their broader applicability. Genetic backgrounds, environmental exposures, sample sizes, and lifestyle factors can significantly influence immune system dynamics, potentially limiting the generalizability of our findings. This underscores the importance of conducting similar studies across large and diverse populations to identify universal patterns and population‐specific variations in immune system development. Looking forward, expanding the scope of such research to include a wider age range could provide a more comprehensive understanding of immune system development across the human lifespan.

In conclusion, our investigation into the IR in a healthy pediatric cohort provides insights into age‐related changes in the immune system. By identifying age‐specific patterns in TCRβ repertoire dynamics, our study contributes to the understanding of immune system development from infancy to adolescence. These findings may inform approaches to vaccination and disease management for children, taking into account the unique immune profiles at different developmental stages. The observed early diversification of the immune system suggests the importance of early life exposures in shaping long‐term immune health. This work sheds light on the future pediatric immunology, potentially guiding the development of age‐appropriate immunological interventions. However, further studies are needed to fully elucidate the clinical implications of these findings and their applicability across diverse populations.

## Author Contributions

M.F., Y.A., and X.Z. designed the research, Y.A., Y.D., Q.Z., X.Q., and L.Z. provided samples and characterization of patient clinical information. Y.M. and M.F. performed data analysis and result visualization. M.F. did the data interpretation and drafted the manuscript. L.Z., Y.M., H.Z., and L.L. performed the TCR sequencing. Y.M., X.Z., and Y.A. revised the manuscript. All authors read and approved the final manuscript.

## Conflicts of Interest

The authors declare no conflicts of interest.

## Supporting information


**Figure S1.** Age‐associated changes in TCRβ repertoire characteristics in the validation dataset. Scatter plots showing the correlation (Spearman’s rank correlation) between age and key TCR repertoire metrics (Shannon diversity index, number of unique clonotypes, number of high‐frequency clonotypes, percentage of in‐frame, percentage of top 100 clonotypes and Gini coefficient) in 25 healthy individuals aged 0–20 years. Each dot represents an individual sample. The red line indicates the linear regression fit, with the shaded area representing the 95% confidence interval.

**Figure S2.**Comparative analysis of TCRβ diversity indices by gender. No significant differences in TCR characteristics were observed between males and females in (A) our primary cohort across different age groups and (B) the validation dataset of 25 healthy individuals aged 0–20 years.

**Figure S3.** Comparison of lengths of deletions of bases of samples from different age subgroups.

**Figure S4.** Abundance of public clonotypes in all samples. (A) Abundance of public clonotypes present in only one age subgroup in all samples. (B–F) Abundance of public clonotypes present in two (B), three (C), four (D), five (E), and six (F) age groups in all samples.

**Figure S5.** Analysis of public clonotype reproducibility and age‐associated characteristics. (A) Table and bar plots showing the reproducibility of public clonotypes identified in our study within the validation dataset. (B) Clustering propensity of public clonotypes across age groups. This panel visualizes the T‐cell receptor clustering using the GLIPH algorithm for different age groups. Each cluster diagram illustrates how public clonotypes tend to group together, indicating a more focused receptor repertoire in younger ages. (C) Comparative analysis of CDR3 sequence lengths. This graph displays the distribution of CDR3 lengths for public clonotypes (in red) versus randomly selected clonotypes (in blue) across the same age groups. Notably, public clonotypes consistently show shorter lengths, highlighting their evolutionary conservation. Statistical significance is indicated above each pair of bars, illustrating substantial differences across age groups, particularly in early childhood.

## Data Availability

The data that support the findings of this study are available on request from the corresponding author. The data are not publicly available due to privacy or ethical restrictions.
