# Supplementary figures and images for: Age‐Related Dynamics and Spectral Characteristics of the TCRβ Repertoire in Healthy Children: Implications for Immune Aging
Source: Aging Cell. 2025 Jan 2;24(4):e14460. doi: 10.1111/acel.14460 (PMC11984678; doi:10.1111/acel.14460)

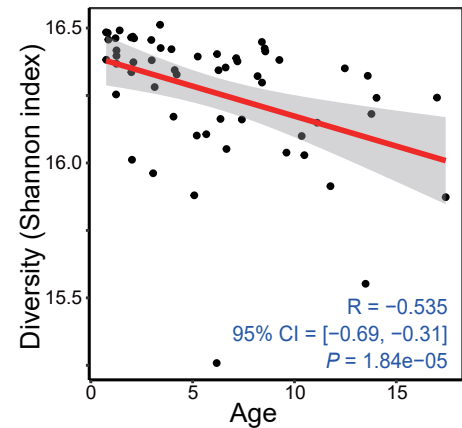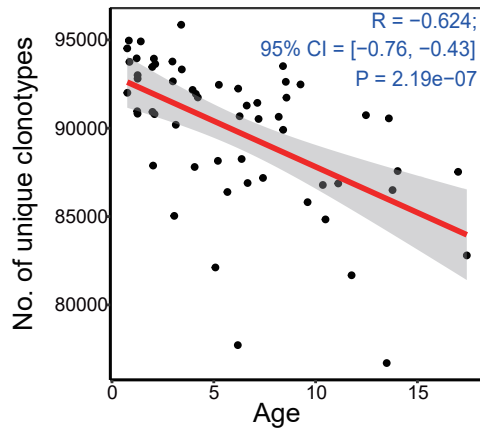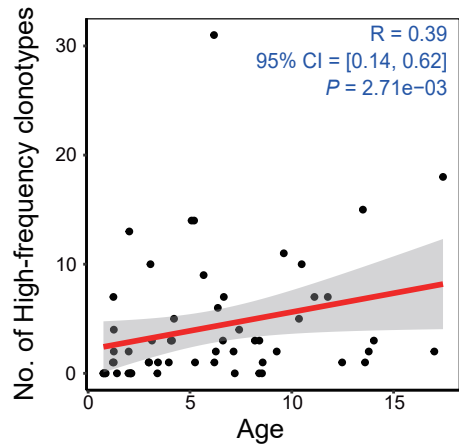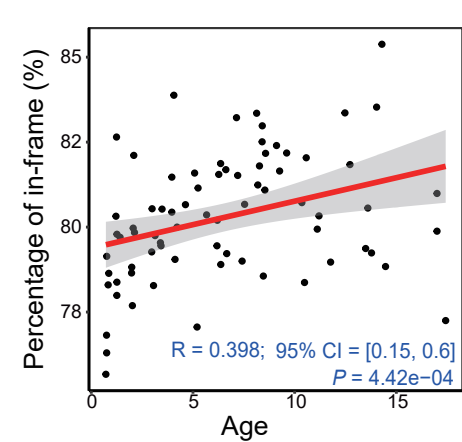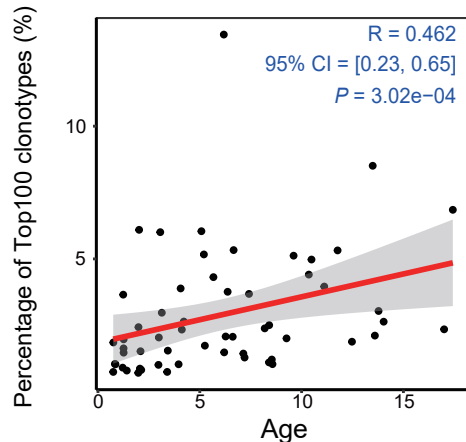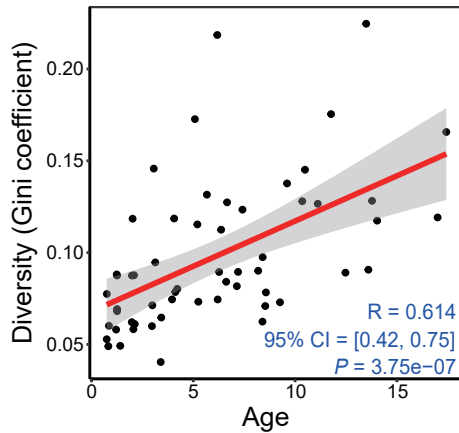

Supplement: Supplementary file 1 — Figure S1. Age‐associated changes in TCRβ repertoire characteristics in the validation dataset. Scatter plots showing the correlation (Spearman’s rank correlation) between age and key TCR repertoire metrics (Shannon diversity index, number of unique clonotypes, number of high‐frequency clonotypes, percentage of in‐frame, percentage of top 100 clonotypes and Gini coefficient) in 25 healthy individuals aged 0–20 years. Each dot represents an individual sample. The red line indicates the linear regression fit, with the shaded area representing the 95% confidence interval. [file ACEL-24-e14460-s002.pdf]

A

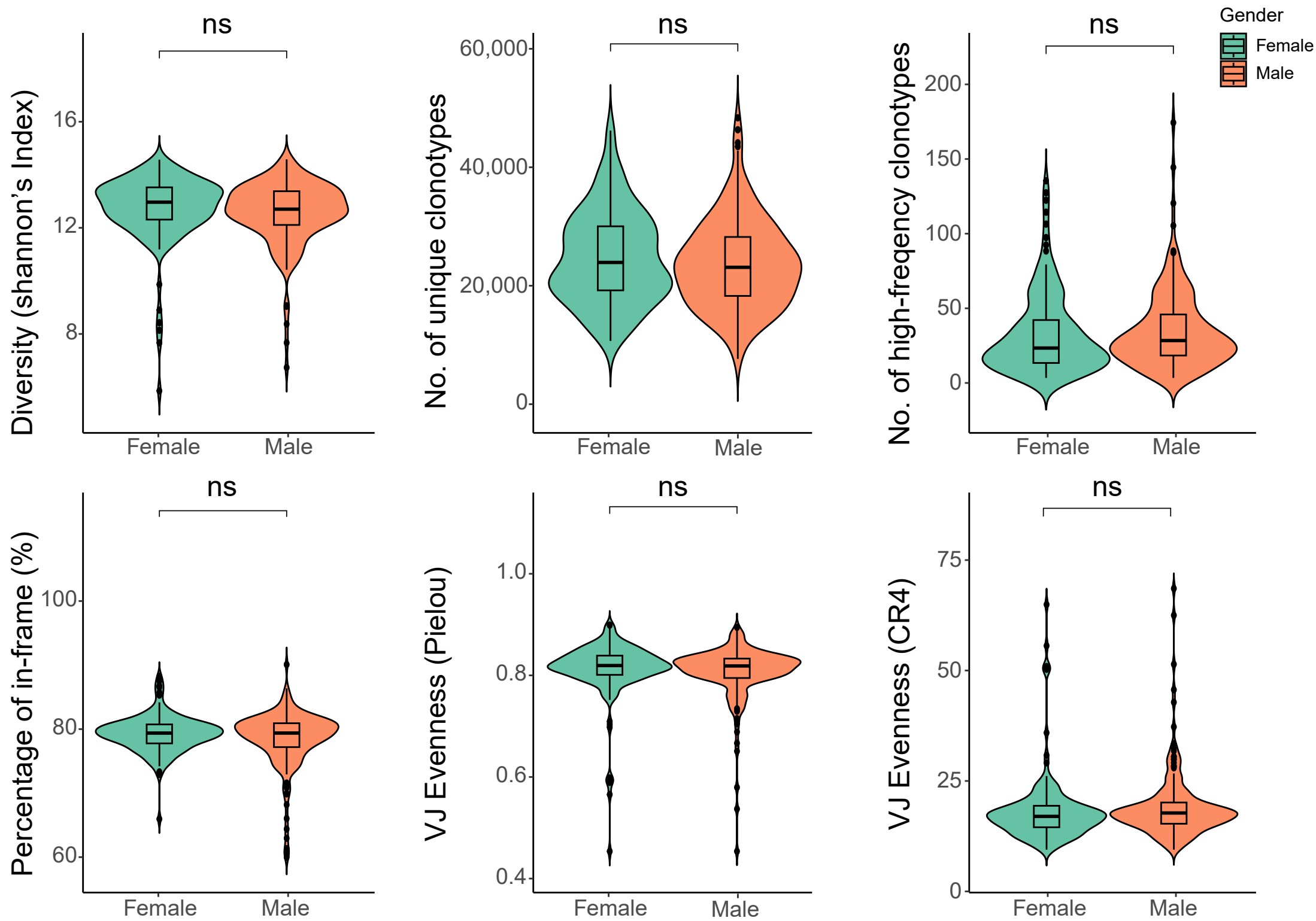

B

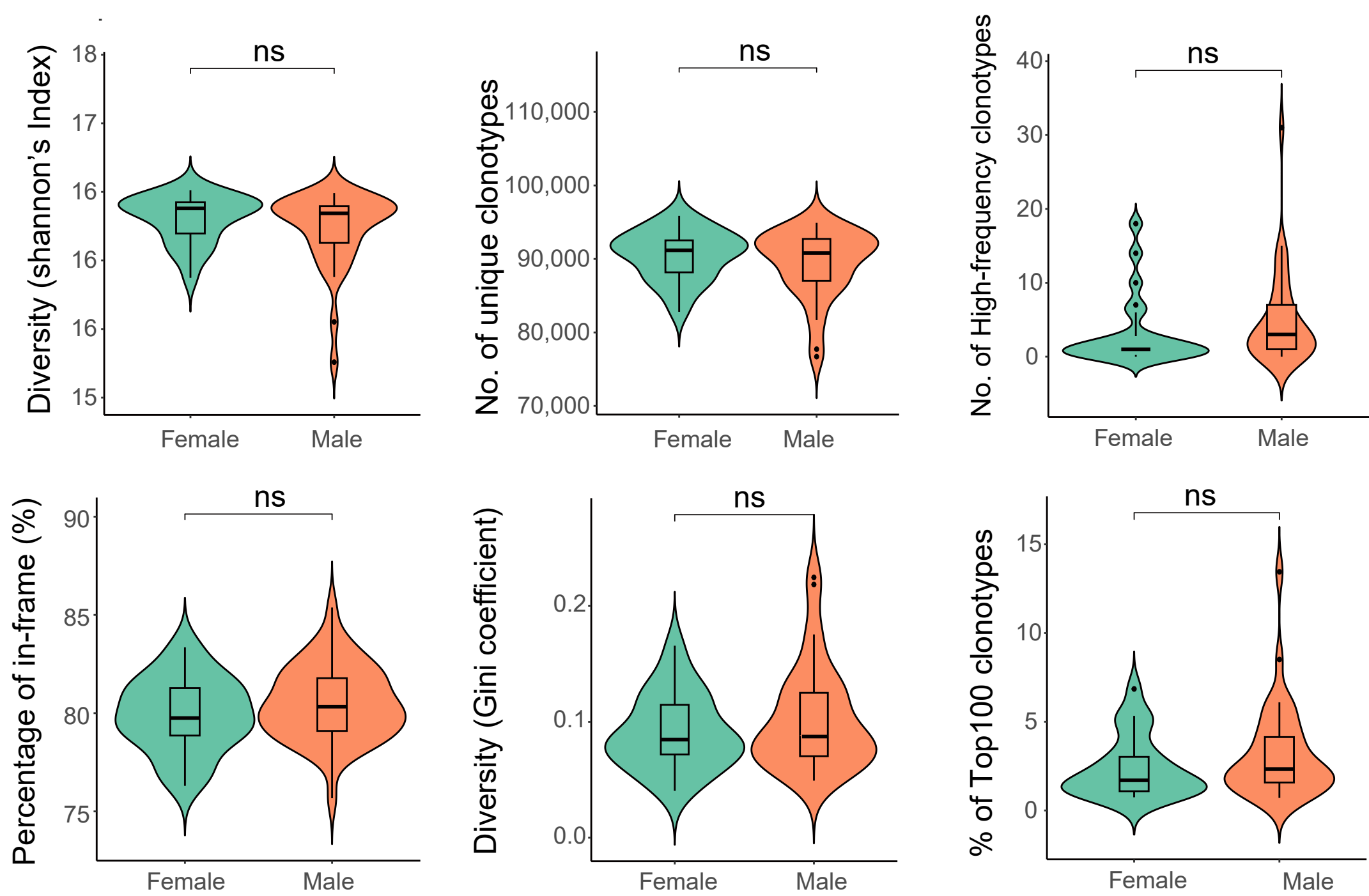

Supplement: Supplementary file 2 — Figure S2.Comparative analysis of TCRβ diversity indices by gender. No significant differences in TCR characteristics were observed between males and females in (A) our primary cohort across different age groups and (B) the validation dataset of 25 healthy individuals aged 0–20 years. [file ACEL-24-e14460-s005.pdf]

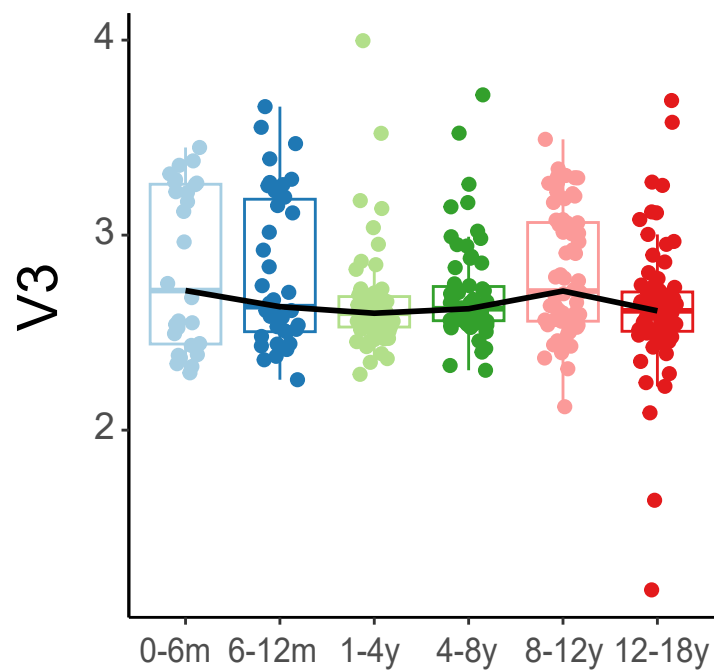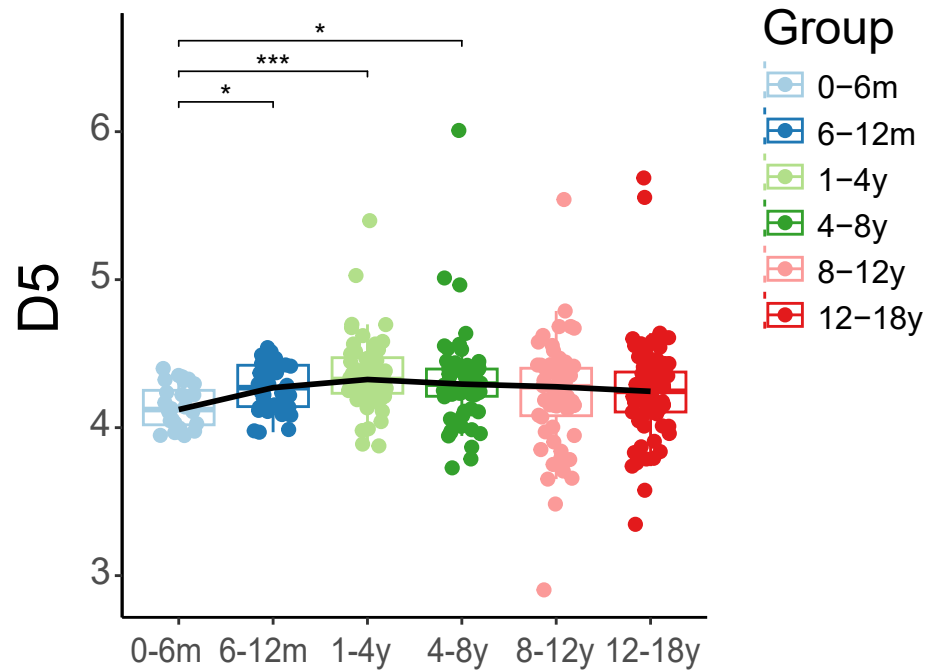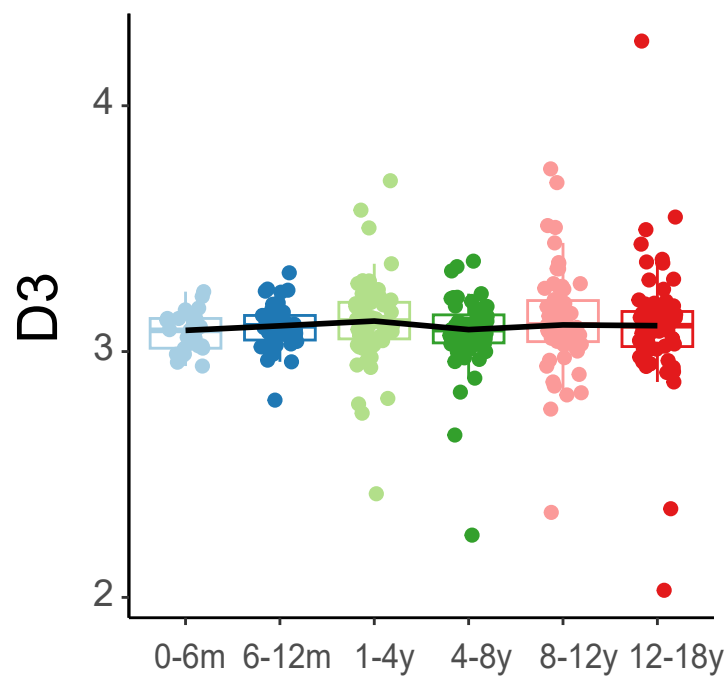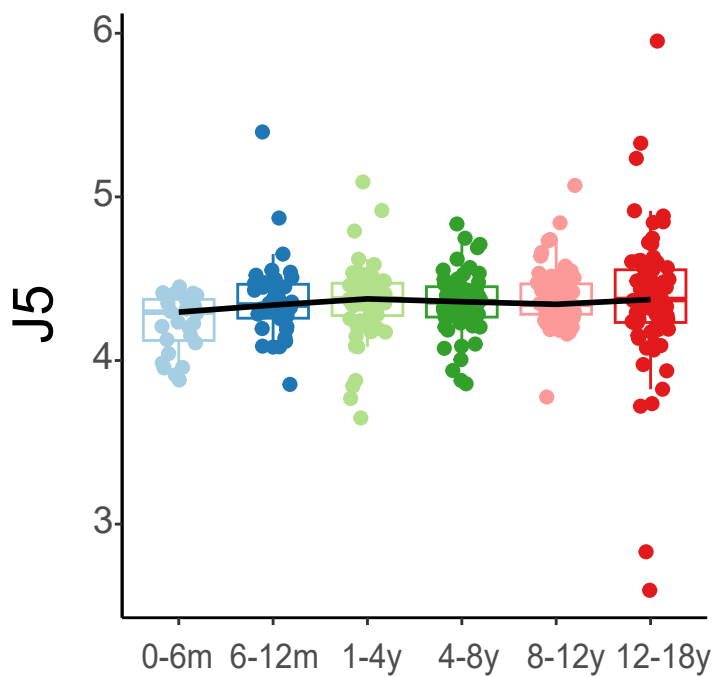

Supplement: Supplementary file 3 — Figure S3. Comparison of lengths of deletions of bases of samples from different age subgroups. [file ACEL-24-e14460-s004.pdf]

**A**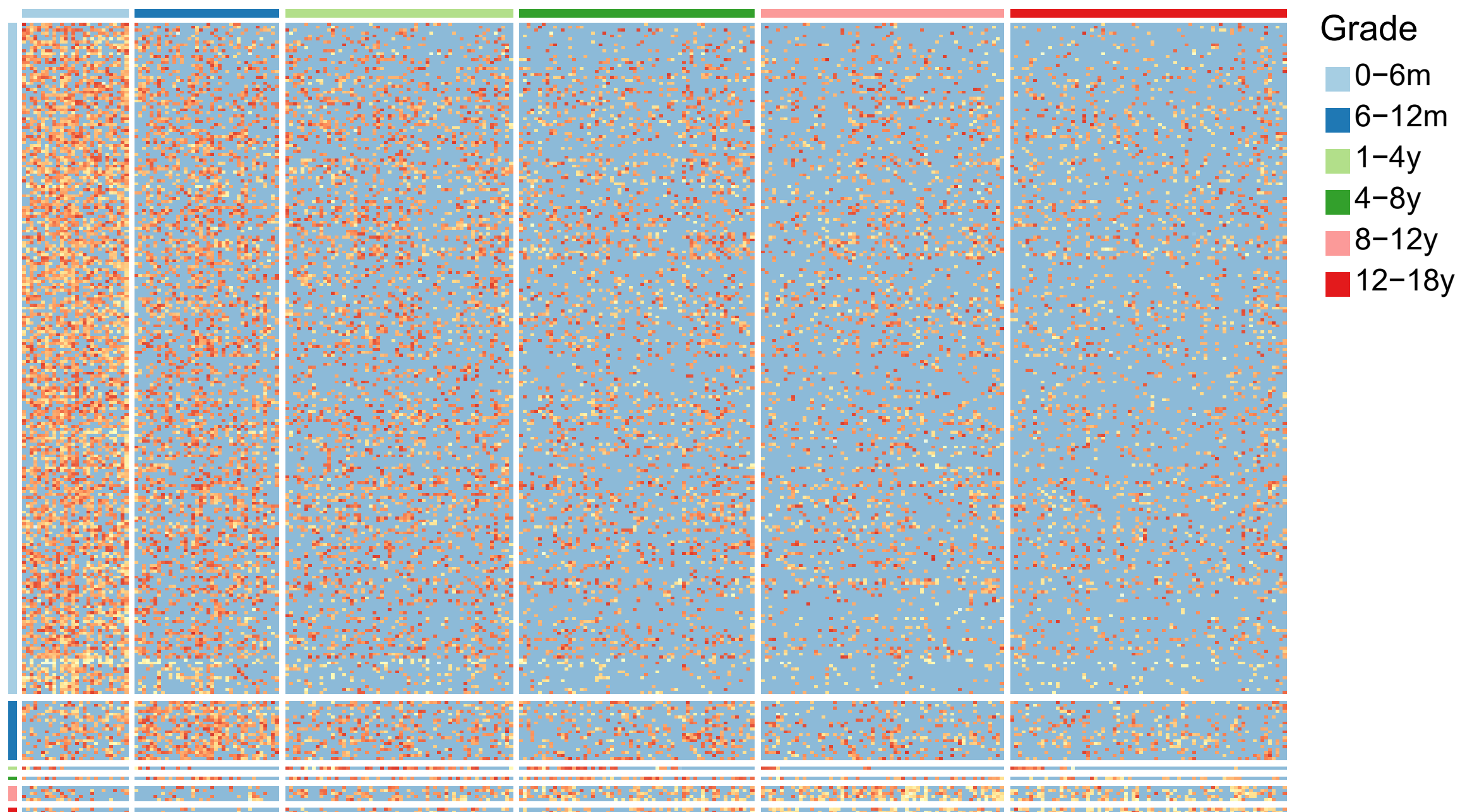**B**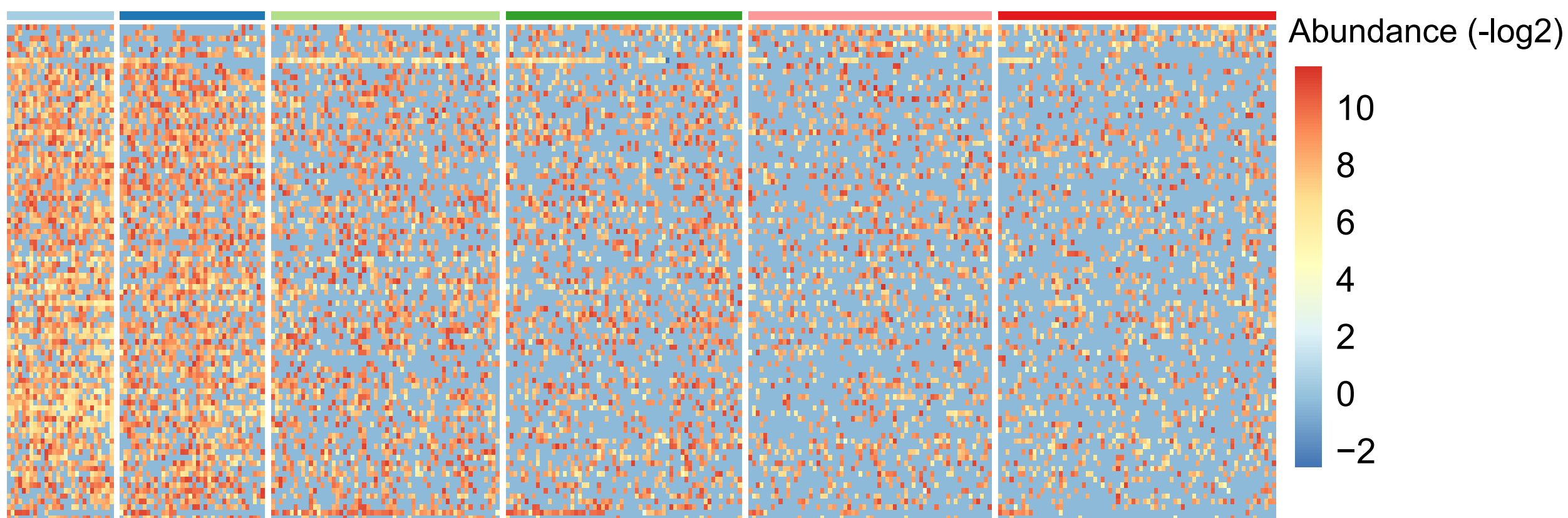**C**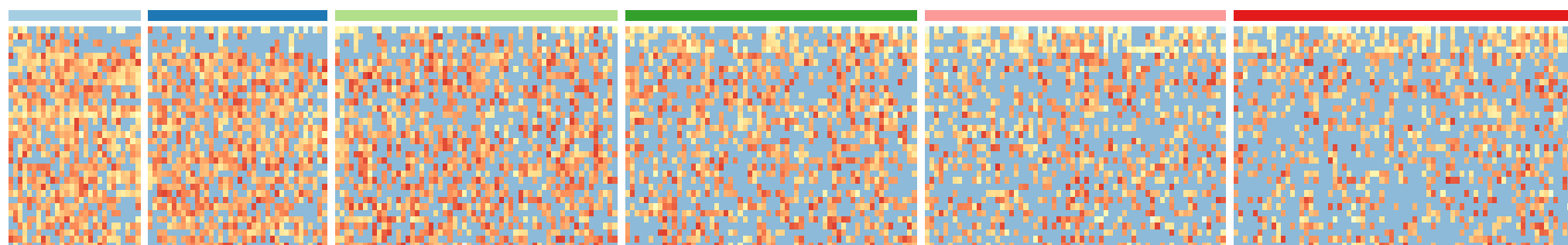**D**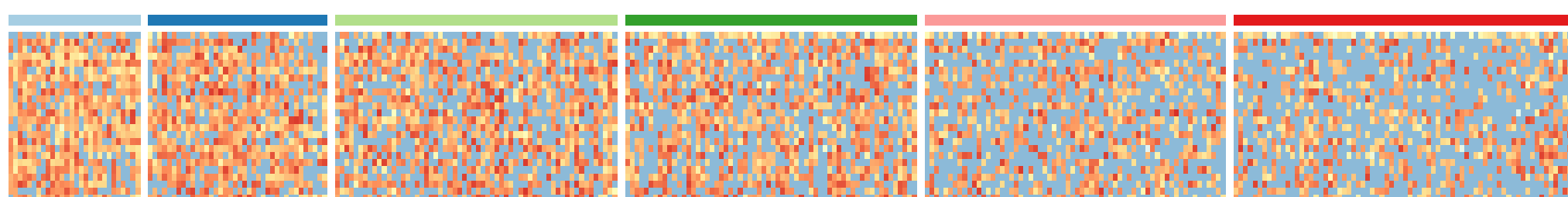**E**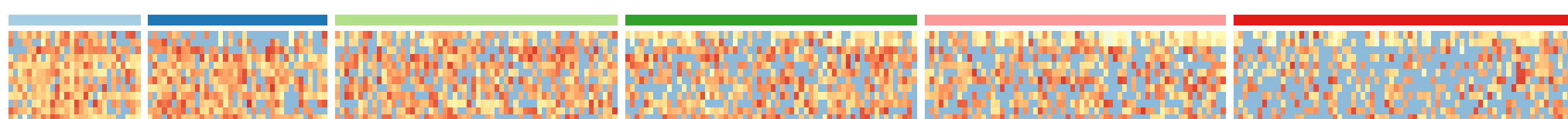**F**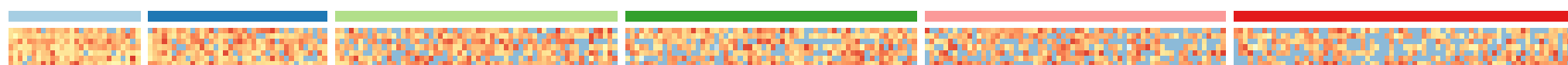

Supplement: Supplementary file 4 — Figure S4. Abundance of public clonotypes in all samples. (A) Abundance of public clonotypes present in only one age subgroup in all samples. (B–F) Abundance of public clonotypes present in two (B), three (C), four (D), five (E), and six (F) age groups in all samples. [file ACEL-24-e14460-s001.pdf]
