# Supplementary material for: Age‐Related Dynamics and Spectral Characteristics of the TCRβ Repertoire in Healthy Children: Implications for Immune Aging
Source: Aging Cell. 2025 Jan 2;24(4):e14460. doi: 10.1111/acel.14460 (PMC11984678; doi:10.1111/acel.14460)

A

Age_group	No. of samples in current study	No. of Public clonotypes in current study	Reproducible_count	Reproducibility_rate
6−12m	37	170	168	98.82%
1−4y	29	77	74	96.1%
4−8y	60	59	57	96.61%
8−12y	62	37	35	94.59%
12−18y	64	23	22	95.65%

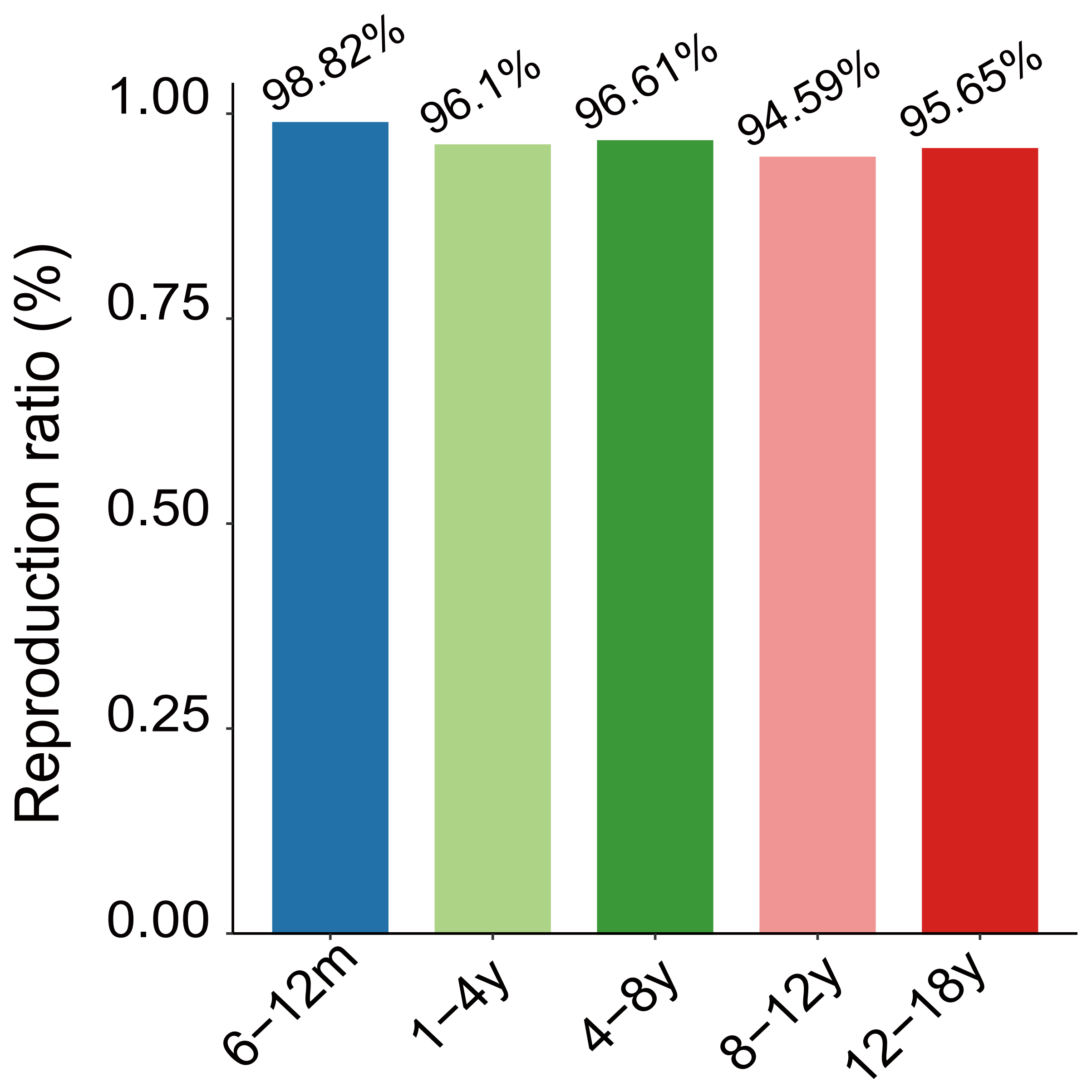

B

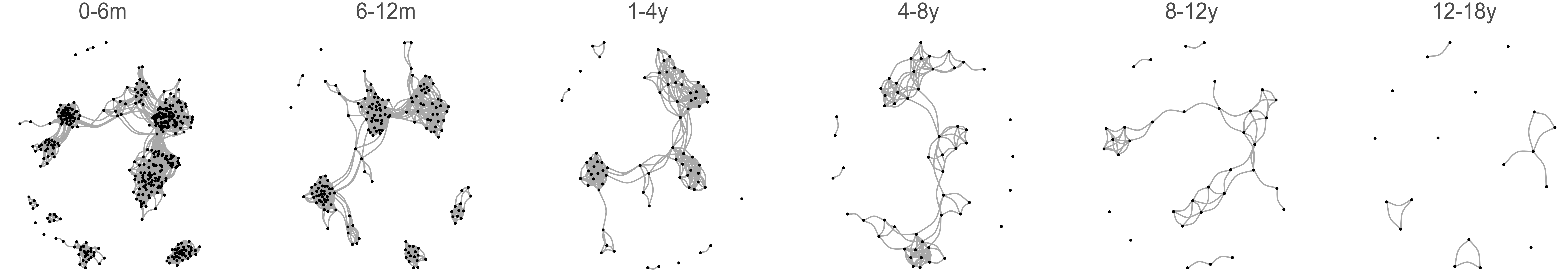

C

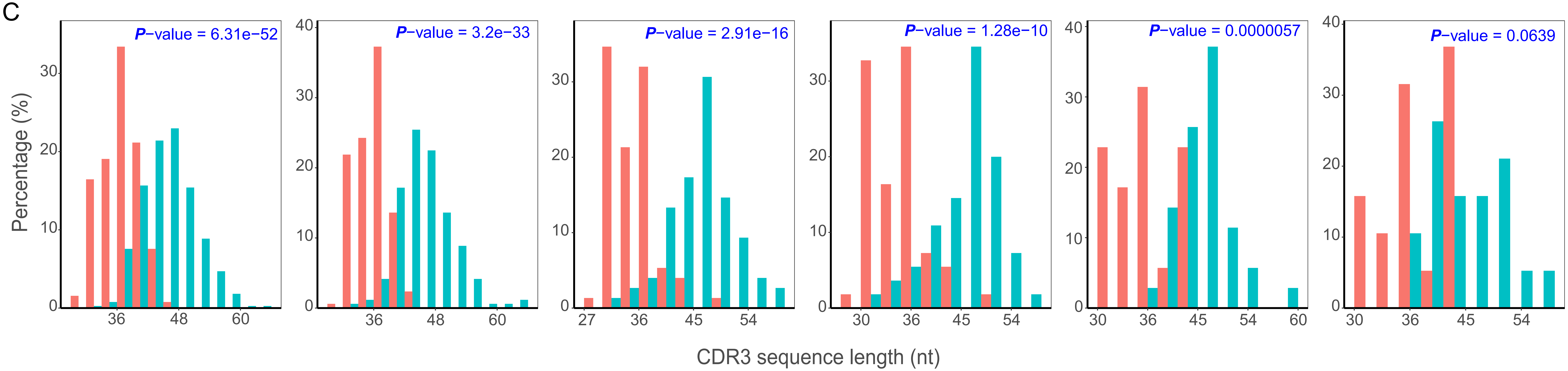

Supplement: Supplementary file 5 — Figure S5. Analysis of public clonotype reproducibility and age‐associated characteristics. (A) Table and bar plots showing the reproducibility of public clonotypes identified in our study within the validation dataset. (B) Clustering propensity of public clonotypes across age groups. This panel visualizes the T‐cell receptor clustering using the GLIPH algorithm for different age groups. Each cluster diagram illustrates how public clonotypes tend to group together, indicating a more focused receptor repertoire in younger ages. (C) Comparative analysis of CDR3 sequence lengths. This graph displays the distribution of CDR3 lengths for public clonotypes (in red) versus randomly selected clonotypes (in blue) across the same age groups. Notably, public clonotypes consistently show shorter lengths, highlighting their evolutionary conservation. Statistical significance is indicated above each pair of bars, illustrating substantial differences across age groups, particularly in early childhood. [file ACEL-24-e14460-s003.pdf]
